# A human protein hydroxylase that accepts *D*-residues

**DOI:** 10.1038/s42004-020-0290-5

**Published:** 2020-05-01

**Authors:** Hwanho Choi, Adam P. Hardy, Thomas M. Leissing, Rasheduzzaman Chowdhury, Yu Nakashima, Wei Ge, Marios Markoulides, John S. Scotti, Philip A. Gerken, Helen Thorbjornsrud, Dahye Kang, Sungwoo Hong, Joongoo Lee, Michael A. McDonough, Hwangseo Park, Christopher J. Schofield

**Affiliations:** 1 Chemistry Research Laboratory, Department of Chemistry, University of Oxford, 12, Mansfield Road, Oxford, OX1 3TA UK; 2 Department of Bioscience and Biotechnology, Sejong University, 209 Neungdong-ro, Kwangjin-gu, Seoul, 05006 Korea; 3 Center for Catalytic Hydrocarbon Functionalizations, Institute for Basic Science (IBS), Daejeon, 34141 Korea; 4 Department of Chemistry, Korea Advanced Institute of Science and Technology (KAIST), Daejeon, 34141 Korea; 5 Department of Chemical and Biological Engineering, Northwestern University, 2145 Sheridan Road, Evanston, IL 60208 USA

**Keywords:** Biocatalysis, X-ray crystallography

## Abstract

Factor inhibiting hypoxia-inducible factor (FIH) is a 2-oxoglutarate-dependent protein hydroxylase that catalyses C3 hydroxylations of protein residues. We report FIH can accept (*D*)- and (*L*)-residues for hydroxylation. The substrate selectivity of FIH differs for (*D*) and (*L*) epimers, e.g., (*D*)- but not (*L*)-allylglycine, and conversely (*L*)- but not (*D*)-aspartate, undergo monohydroxylation, in the tested sequence context. The (*L*)-Leu-containing substrate undergoes FIH-catalysed monohydroxylation, whereas (*D*)-Leu unexpectedly undergoes dihydroxylation. Crystallographic, mass spectrometric, and DFT studies provide insights into the selectivity of FIH towards (*L*)- and (*D*)-residues. The results of this work expand the potential range of known substrates hydroxylated by isolated FIH and imply that it will be possible to generate FIH variants with altered selectivities.

## Abstract (web summary)

Hypoxia-inducible factor (FIH) is an oxygenase which post-translationally hydroxylates proteins and is implicated in a range of biological processes. Here a wide substrate tolerance for FIH is demonstrated, including for d-amino acids, where double hydroxylation of d-leucine is observed.

## Introduction

**Fig. 1:**
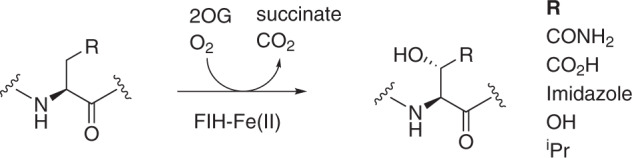
Reactions catalysed by FIH. FIH is a 2OG oxygenase catalyzing the posttranslational C3 hydroxylation of (*L*)-residue substrates including the hypoxia-inducible factor α-subunits (HIFα; *R* = CONH_2_) and ankyrin repeat domain proteins (R = CONH_2_, COOH, OH, imidazole and isopropyl) to give mono-hydroxylated products. Note all studied (*L*)/(*2S*)-substrates give products with the same relative stereochemistry at C3, i.e., (*2S,3S*) for R = CONH_2_, COOH, Imidazole, OH or (*2S,3R*) for R = ^i^Pr.

Biopolymer hydroxylation as catalysed by Fe(II) and 2-oxoglutarate (2OG)-dependent oxygenases^1,2^ is important in biological processes including oxygen sensing^3–7^, chromatin regulation, carnitine biosynthesis, lipid metabolism, collagen biosynthesis, and DNA/RNA modification/repair^2,7–10^. The 2OG oxygenase factor inhibiting hypoxia-inducible factor (FIH) is of particular interest from an enzymology selectivity perspective, because it catalyses the posttranslational hydroxylation of multiple protein residues^11–15^. FIH was first identified as an asparaginyl C3 hydroxylase acting on hypoxia-inducible factor α-subunits; such modification reduces the transcriptional activity of HIF in a manner relevant to the hypoxic response in animals^16–20^. FIH also catalyses C3 hydroxylation of residues on multiple human members of the ubiquitous ankyrin repeat domain (ARD) protein family^12–15,21–23^. In the case of the ARD proteins, FIH catalyses hydroxylation not only of Asn-residues but also of Asp- and His-residues^12,14,15^. More recent work has shown that FIH can catalyse the hydroxylation of residues with hydrophobic side chains, such as leucine, to form C3 hydroxylated products with the same relative stereoselectivity as in the hydroxylation of residues with polar side chains, i.e., to form the (3*S*)-hydroxylated product (Fig. 1)^12^.

Here we report that FIH can catalyse the hydroxylation of (*D*)-residues, interestingly with altered substrate and product selectivities compared with the analogous (*L*)-residues. Unexpectedly, in the case of the (*D*)-Leu residue substrate, a dihydroxylated product is observed.

## Results

### FIH hydroxylation assays with *D*-residue containing peptides

The promiscuity of some 2OG oxygenase protein hydroxylases is precedented by earlier work on 2OG oxygenases acting on small molecules, including those involved in β-lactam biosynthesis^24,25^. In some cases, unsaturated substrate analogues were found to undergo rearrangements, in some examples likely via radical intermediates^26,27^. Inspired by these studies, we tested whether FIH can catalyse hydroxylation of an allylglycine residue; anticipating hydroxylation of allylglycine might be more efficient than for leucine due to its relatively weak C3 allylic C–H bond, or that 1,3-allylic rearrangement or epoxide formation may occur, as precedented for 2OG oxygenases catalyzing small molecule oxidations^9^. A consensus ARD peptide substrate^22,23^ for FIH with an allylglycine residue at the potential hydroxylation position was therefore prepared. For synthetic efficiency reasons, the peptide was initially produced with an epimeric (*D*/*L*) mixture of allylglycine at the anticipated potential hydroxylation position. When incubated with FIH under standard conditions^22,23^, the (*D*/*L*) epimeric mixture reproducibly manifested ~50% conversion to a hydroxylated product; as evidenced by a +16 Da mass increment observed by matrix-assisted laser desorption ionization time-of-flight (MALDI-ToF) mass spectrometry (MS) analysis. To investigate further, the corresponding peptide with an (*L*)-allylglycine residue at the hydroxylation position was made. Surprisingly, given the extensive evidence for FIH-catalysed (*L*)-residue hydroxylation, this peptide did not undergo hydroxylation (<5%) upon incubation with FIH. By contrast, the subsequently synthesized (*D*)-allylglycine peptide underwent relatively efficient hydroxylation, demonstrating that FIH can, and at least in this case prefers to, catalyse hydroxylation of (*D*)-residues (Supplementary Fig. 1g).

To investigate the scope of FIH-catalysed hydroxylation of (*D*)-residue containing substrates, systematic substitutions at the hydroxylation position of the consensus ankyrin (CA) scaffold peptide^22,23^ (i.e., HLEVVKLLLEHGADVXAQDK where X is the substituted residue) were then made. The results (Supplementary Fig. 1 and Supplementary Table 1) show that almost half of the *D*-residue containing peptides tested (9/19) manifested apparent oxidation in the presence of FIH. An approximate order of efficiency of hydroxylation under standard conditions are (*D*)-Leu ≈ (*L*)-Asn > (*D*)-Ile > (*D*)-Asn ≈ (*D*)-AllylGly > (*D*)-His ≈ (*D*)-Phe ≈ (*D*)-Try > (*D*)-Phe > (*D*)-Trp (Supplementary Table 1b). (*D*)-Residue substrates with amide, basic, nucleophilic, aromatic, and hydrophobic side chains, were identified indicating FIH may accept an even wider range of (*D*)- than (*L*)-residues^12^. Differences in the selectivity for (*D*)-residues were apparent with (*D*)-Phe and (*D*)-Tyr being apparently hydroxylated by FIH, whereas the (*L*) version of these peptides were not hydroxylated. Further, the (*D*)-Leu, (*D*)-Ile, and (*D*)-Trp-containing peptides were apparently oxidized more efficiently than their (*L*)-counterparts^12^. By contrast, the (*D*)-Asp CA peptide was not hydroxylated, whereas the (*L*)-Asp peptide was^12^. The results also suggested (*D*)-Cys- and (*D*)-Met-containing CA sequences may be substrates, although these observations require further work due to the readily oxidized sulfur of these substrates.

**Fig. 2:**
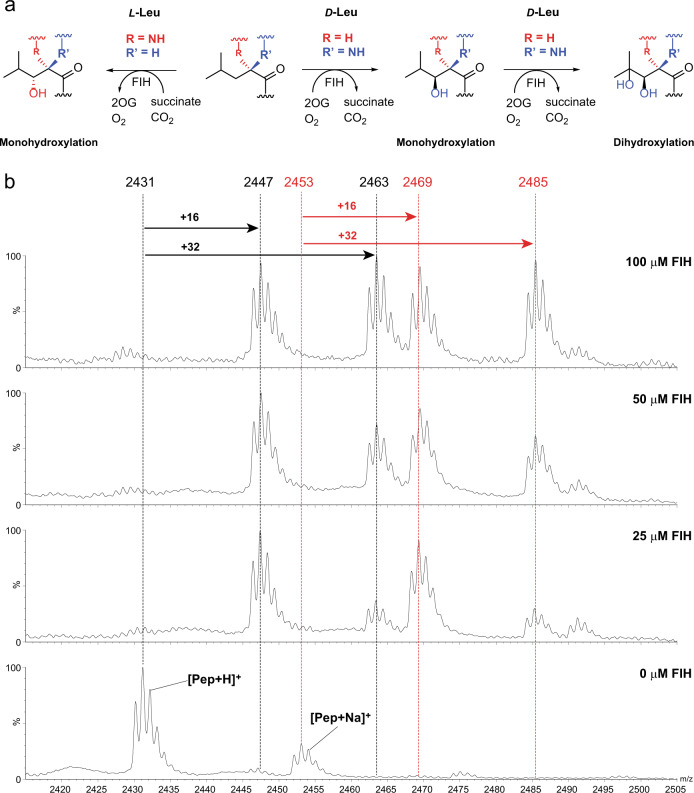
FIH can catalyse dihydroxylation reactions. **a** FIH catalyses single- and double-hydroxylations of (*L*)- and (*D*)-leucine-containing residues, respectively. **b** Mass spectrometric analysis of FIH-catalysed hydroxylation of a (*D*)-leucine residue in a consensus ankyrin (CA) (HLEVVKLLLEHGADV((***D***)-**L**)AQDK) substrate. The CA (*D*)-Leu peptide (50 μM) was incubated with 0 μM, 25 μM, 50 μM and 100 μM FIH. Sub-stoichiometric quantities of FIH result in predominantly mono-hydroxylated product, whereas at higher FIH concentrations the dihydroxylated product is produced. Note the different C3 hydroxylation stereochemistries for the (*L*)-Leu and (*D*)-Leu substrates. See text for discussion.

Most unexpectedly, the (*D*)-Leu CA peptide appeared to undergo a double hydroxylation, with both +16 Da and +32 Da peaks being significant in the MS spectrum after incubation, albeit with high levels of FIH (Fig. 2a). In the case of the (*L*)-Leu CA peptide substrate, only a +16 Da mass shift was observed, as reported^12^, suggesting a single hydroxylation. To investigate whether the apparent dihydroxylation observed with (*D*)-Leu CA peptide reflects 1,2 or 1,3 diol formation, the high-performance liquid chromatography (HPLC)-purified product peptide was treated with NaIO_4_, which reacts with 1,2-diols (Supplementary Fig. 2)^28^. Partial cleavage of the apparent dihydroxylated product (2462.8 Da) to give a product of mass 2402.7 Da was observed, consistent with 1,2- and not 1,3- oxidation to give a C3 aldehyde (Supplementary Fig. 2). Addition of biotin hydrazide to the reaction mixture gave a new product with a +212 Da increment, again consistent with formation of a C3 aldehyde.

To investigate whether a mono-hydroxylated (*D*)-Leu peptide can undergo further hydroxylation to form the overall +32 Da product, a synthetic CA peptide containing the (2*R*,3*S*) 3-hydroxyleucine residue was assayed. This peptide was observed by MS to be hydroxylated to only a small extent, if at all (much less than the (*D*)-Leu CA peptide) (Supplementary Fig. 1g).

### Calculations on FIH-catalysed dihydroxylation

To investigate the differences in hydroxylation of (*D*)- and (*L*)-leucine-containing CA substrates, we investigated the reactions using density functional calculations (B3LYP/6–31 G** level), using simplified model systems at the ferryl intermediate stage of catalysis (Supplementary Fig. 3). The modelling studies were based on crystallographic analyses on (*D*)- and (*L*)- residue containing FIH substrates as described below.

**Fig. 3:**
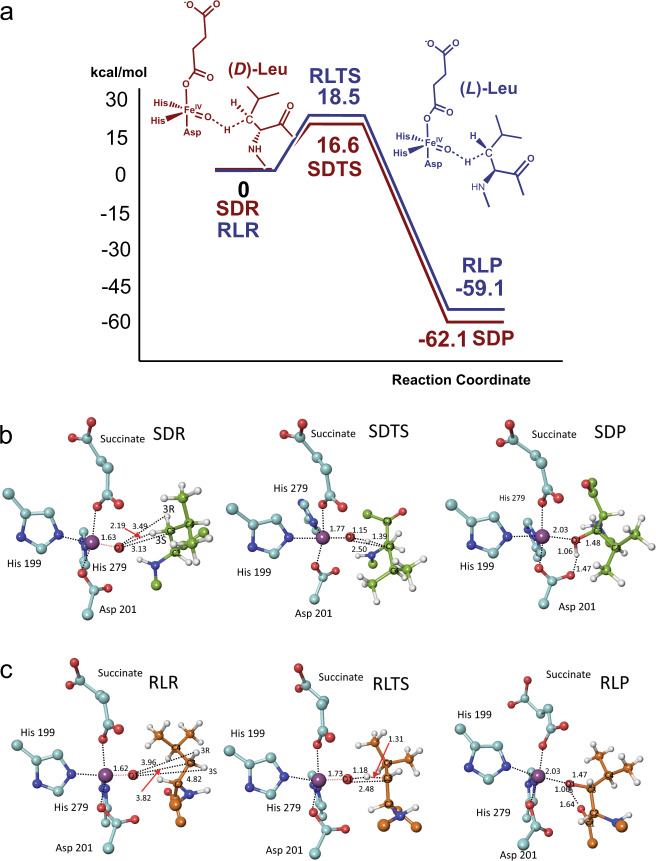
Mechanistic insights into FIH-catalysed C3 hydroxylation of (*D*)- and (*L*)-leucine. **a** Potential energy profile diagrams along the modelled reaction coordinates for the FIH-catalysed hydroxylation of (*D*)-leucine (brown) or (*L*)-leucine (blue)-derived substrate models. The potential energies of stationary-state structures for the (*D*)- and (*L*)-leucine residue substrate hydroxylations are measured from the SDR or RLR intermediate structures, respectively. **b** Optimized structures of energy minima and transition state for C3 hydroxylation of the (*D*)-leucine substrate. **c** Optimized structures of the energy minima and the transition state for C3 hydroxylation of the (*L*)-leucine substrate. Colours: Fe (blue purple), N (blue), O (red), C (turquoise), substrate (*D*)-leucine (yellow-green), (*L*)-leucine (orange), H (white). Selected distances are in Å.

Figure 3b shows calculated structures for FIH complexed with a ‘truncated’ (*D*)-leucine substrate model (i.e., with an N-methylated amino and C-terminal methyl ketone, referred to as (*D*)-Leu) (SDR), the mono-hydroxylated product model (SDP), and the calculated transition state (SDTS)). In SDR, the reactive iron-bound oxygen (O1) is proximate to (*D*)-Leu C3, being ∼2.2 Å from the *pro-S* hydrogen. By contrast, the *pro-R* (*D*)-Leu C3 hydrogen projects away from O1 (∼3.5 Å), suggesting formation of the (2*R*,3*S*) mono-hydroxylated product (as observed—see below).

When the O1**···**C3 distance contracts from 3.1 Å (in SDR) to 2.5 Å, a transition state (SDTS) manifests, wherein the *pro-S* hydrogen is partially transferred to O1. The O**···**H distance decreases from 2.2 Å in SDR to 1.2 Å in SDTS, corresponding to >90% formation of alcohol product (SDP). Despite investigations, there was no evidence for carbanionic or radical intermediates, suggesting an efficient radical rebound type mechanism (Fig. 3a), as proposed for other 2OG oxygenases^29–33^. In SDP, the newly formed alcohol points towards the Asp201 carboxylate, forming a hydrogen bond (1.5 Å).

With (*L*)-Leu, the model implies C3 *pro-R* hydroxylation; because the distance between O1 and C3 is increased from 3.1 Å in SDR to 3.8 Å in RLR, a higher activation barrier is predicted for (*L*)- compared with (*D*)-Leu hydroxylation, consistent with the experimental observations. The calculations imply hydroxylation of both (*D*)- and (*L*)-Leu is thermodynamically favourable; SDP and RLP are both ∼60 kcal/mol lower in potential energy than SDR and RLR (Fig. 3). The calculated activation barrier for hydroxylation of (*D*)-Leu is somewhat lower than that of (*L*)-Leu by 1.9 kcal/mol. It is noteworthy that the role of hydrogen bond acceptor with respect to the product alcohol changes from Asp201 in SDP (Fig. 3b) to the backbone carbonyl oxygen of Leu803 in RLP (Fig. 3c).

**Fig. 4:**
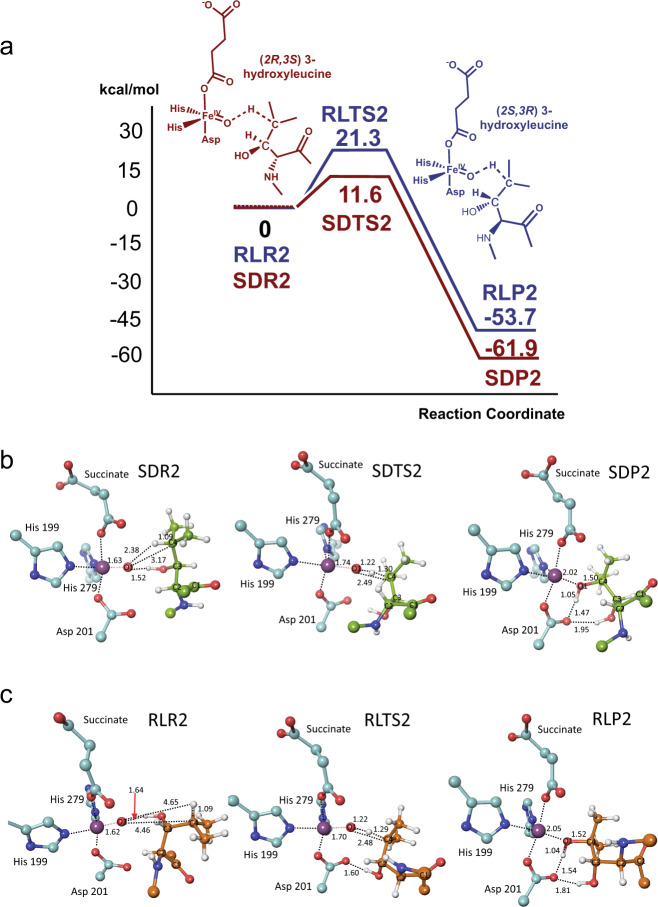
Mechanistic insights into the second FIH-catalysed hydroxylation of (*D*)-leucine. **a** Potential energy profiles for the ‘second’ hydroxylation of (2*R*,3*S*) C3 hydroxyleucine (brown) and (2*S*,3*R*) C3 hydroxyleucine (blue) substrates. The potential energies of stationary-state structures on the reaction coordinates are measured from the respective reactant complexes (SDR2 and RLR2). **b** Optimized structures of energy minima and transition states for the ‘second’ hydroxylation reaction of the (2*R*,3*S*) C3 hydroxyleucine substrate. **c** Optimized structures of the energy minima and the transition state for the ‘second’ hydroxylation reaction of the (2*S*,3*R*) C3 hydroxyleucine substrate. Colours: Fe (blue purple), N (blue), O (red), C (turquoise), substrate (*D*)-leucine (yellow-green), (*L*)-leucine (orange), H (white). Selected distances are in Å.

We then modelled the (potential) second hydroxylation of the mono-hydroxylated (*D*)- and (*L*)-Leu products (B3LYP/6–31 G** level). The model for the proposed reactive (2*R*,3*S*) 3-hydroxyleucine complex (SDR2) is shown in Fig. 4b, with the dihydroxylated product (SDP2) and transition state (SDTS2). In SDR2, the C4 hydrogen points towards the iron-bound O1, consistent with the experimental results implying C3/C4 vicinal diol formation, although O1 is similarly close to C3 (3.09 Å for C3 vs. 3.17 Å for C4). A second hydroxylation at C3 would give an unstable gem diol, for which there is precedent in 2OG oxygenase catalysis^34^; however, the C3 hydrogen is directed away from O1 in SDR2. Thus, these observations are consistent with the second hydroxylation occurring at C4.

As the reaction proceeds, the hydrogen-bond acceptor with respect to the substrate C3 alcohol changes from O1 to the Asp201 carboxylate. Contributions to the single imaginary frequency in SDTS2 come from the proton transfer from C4 to O1 and from the O1−C4 bond-forming motion. This implies that hydrogen abstraction and O1−C4 product bond formation occur efficiently. The O1**···**H distance contracts from 2.38 to 1.22 Å on going from SDR2 to SDTS2, corresponding to ∼85% completion of O1−H bond formation. By contrast, O1 approaches C4 by only 0.69 Å during formation of SDTS2, with ∼40% progress towards the β,γ-dihydroxy-(*D*)-leucine product.

Consistent with the lack of its experimentally observed second hydroxylation, the modelling results for (2*S*,3*R*) 3-hydroxyleucine differ from those for (2*R*,3*S*) 3-hydroxyleucine (Fig. 4c). In RLR2, C4 is relatively distant from O1 compared with SDR2; the C4**···**O1 distance is 4.5 Å in RLR2 compared with 3.2 Å in SDR2 (Fig. 4). In both SDR2 and RLR2 (Fig. 4), the mono-hydroxylated substrate C3 alcohol can hydrogen bond with O1. The O–H···O hydrogen bond in SDR2 contributes to sustaining a reactive conformation. By contrast, the corresponding hydrogen bond in RLR2 apparently serves as an obstacle to reaction by stabilizing a non-productive conformation.

Despite the difference in activation barriers and reaction energies, most structural features of SDTS2 and SDP2 are similar to those of RLTS2 and RLP2. Differences in distances involving O1 are small and RLTS2 is similar to SDTS2 in forming a hydrogen bond between the substrate alcohol and Asp201 (Figs. 3 and 4). The C3 hydroxyl is a hydrogen bond donor to O1 in both complexes. In SDR2, this contributes to sustaining a reactive conformation of (2*R*,3*S*) 3-hydroxyleucine. Although also present in RLR2, the substrate cannot adopt such a favourable conformation due to the effect of the (2*S*)-sterochemistry on side chain conformation.

The dihydroxy-(*D*)-leucine product (SDP2) is produced with a calculated potential energy of ~62 kcal/mol below the reactant complex (SDR2), indicating that a second hydroxylation reaction with the (2*R*,3*S*) 3-hydroxyleucine substrate is thermodynamically favourable (Fig. 4a); the calculated activation barrier to form the product via SDTS2 is 11.6 kcal/mol, which is 9.7 kcal/mol lower than for (2*S*,3*R*) 3-hydroxyleucine. Thus, on the basis of kinetic and thermodynamic grounds and consistent with the experimental observations, the calculations imply the second hydroxylation is likely more favourable for (2*R*,3*S*)-hydroxyleucine compared with (2*S*,3*R*)-hydroxyleucine.

### FIH crystal structures with *D*-residue containing peptides

**Fig. 5:**
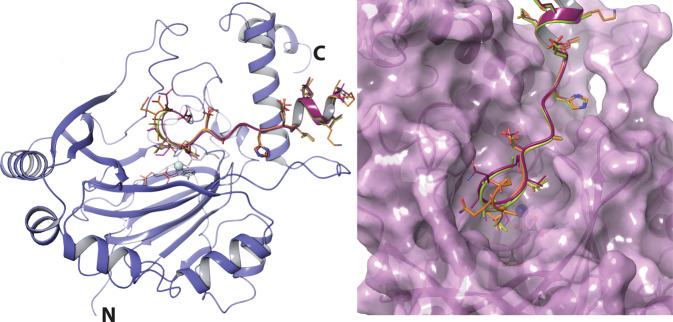
FIH binds peptides with different substrate residues in a similar overall manner. Ribbons representation of superimposed peptide substrates when bound to FIH as observed by X-ray crystallography (violet); orange, (*L*)-leucine (PDB: 4B7E); yellow-green, (*D*)-leucine CA substrate (PDB: 4JAA); maroon, (2*R*,3*S*) C3 hydroxyleucine CA substrate (PDB: 6RUJ); purple, (*D*)-allylglycine CA substrate (PDB: 4NR1). The active site metal is a turquoise sphere. Note only one monomer of the FIH dimer is shown.

**Fig. 6:**
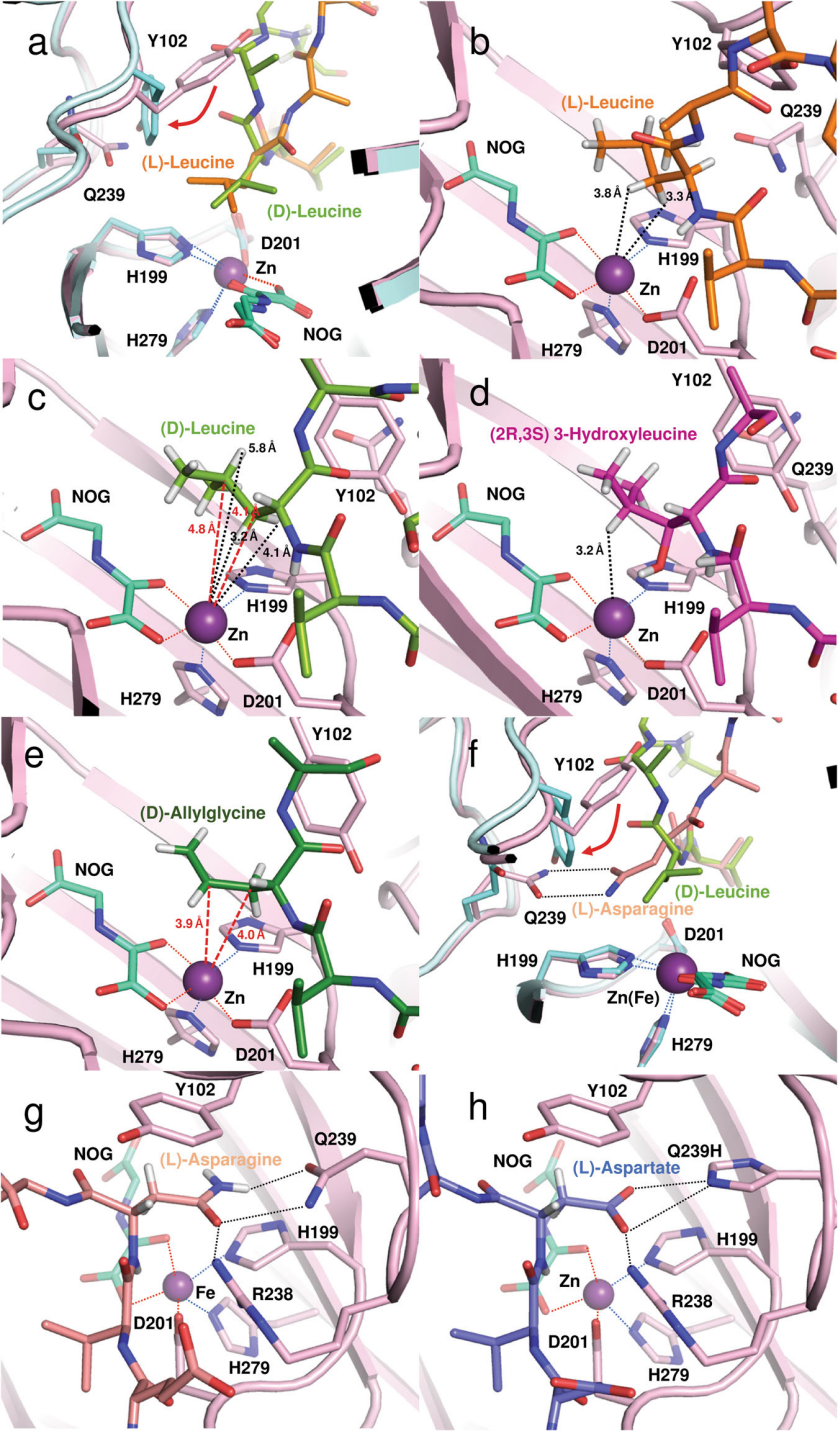
Superposition of views from crystal structures of FIH in complex with consensus ankyrin sequence peptides. Superimposed structures of FIH with: **a** (*L*)-leucine (PDB: 4B7E; orange) and (*D*)-leucine (PDB: 4JAA; yellow-green) residues at the hydroxylation position. FIH atoms close to the substrates are in pink and cyan, for the (*L*)- and (*D*)-substrate views, respectively; **b** (*L*)-leucine; **c** (*D*)-leucine; **d** (2*R*,3*S*) 3-hydroxyleucine (PDB: 6RUJ; purple); **e** (*D*)-allylglycine (PDB: 4NR1; dark-green); **f** (*L*)-asparagine (salmon) and (*D*)-leucine (yellow-green); **g** (*L*)-asparagine (salmon) and **h** (*L*)-aspartate (PDB: 2XUM); slate-blue). Note that hydrogens are shown on the side chains of the substrate residues in the non-superimposed structures.

To investigate the structural basis for the differential selectivities of (*D*)- over (*L*)-residues reported here, we attempted X-ray crystallographic analysis of FIH with (*D*)-substrates, to compare with the analogous (*L*)-substrates. Structures of FIH complexed with (*L*)-Leu, (*D*)-Leu, (2*R*,3*S*) 3-hydroxyleucine and (*D*)-allylglycine CA peptides were obtained (Fig. 5, Supplementary Table 2 and Supplementary Fig. 4). We were unable to obtain a structure with (*L*)-allylglycine CA, which was not observed to be a substrate. The general binding modes of the (*D*)-substrates were similar to those reported for other (*L*)-FIH substrates^12,14,15,20,21,23^. However, based on the available crystal structures and modelling studies, there are differences in the binding modes of the (*L*)- and (*D*)-substrates in the active site region close to the iron. Compared with (*L*)-residue substrate binding, the (*D*)-residues adopt a distinctive orientation that appears to involve rotation about the Cα-Cβ bond of FIH Tyr102 (Fig. 6a–e).

In the case of the (*D*)-allylglycine substrate structure, the side chain does not appear tightly bound, although C3 hydroxylation is likely, C4 is closer than C3 to the active site iron (3.9 Å) (Fig. 6e). Modelling of the (*L*)-allylglycine substrate complex based on this structure implies its allyl group will project away from the metal, consistent with its observed lack of reaction. By contrast, the side chains of substrates with polar side chains (both (*L*) and (*D*)) are positioned to form hydrogen bonds/polar interactions with the side chains of Arg238 and Gln239, as observed in prior FIH-substrate complexes^12,14,15,20^ (Fig. 6g, h). The (*L*)-Leu substrate structure implies that conformational movement of the (*L*)-Leu isopropyl group may be relatively restricted because of close contacts with the Tyr102, His199 and Gln239 side chains of FIH (Fig. 6b). By contrast with the (*L*)-Leu substrate, with  (*D*)-Leu its isopropyl group projects further away from the protein interior (Fig. 6a). The (*D*)-Leu backbone carbonyl oxygen and the neighbouring Ala of the (*D*)-Leu CA peptide substrate appear to push the Tyr102 side chain away from the active site (Fig. 6c). Collectively, these observations suggest more flexibility in binding of (*D*)-Leu compared with (*L*)-Leu, consistent with the observed second hydroxylation of (*D*)-Leu but not (*L*)-Leu (Fig. 6a–d). The apparently more restricted binding of (*L*)-Leu may also relate to the lack of activity observed with the (*L*)- compared with the (*D*)-allylglycine CA peptide.

Consistent with the calculated SDP structure (Fig. 3), in the (2*R*,3*S*)-hydroxyleucine complex crystal structure, the C3 alcohol of the mono-hydroxylated (2*R*,3*S*) product/intermediate is positioned to hydrogen bond with the FIH Asp201 iron-binding carboxylate (Fig. 6d). This interaction may influence hydroxylation to give the subsequently formed dihydroxylated product.

### Stereochemistry of hydroxylation

The crystallographic analyses lead to the prediction that the product of monohydroxylation of (*L*)-Leu will be (2*S*,3*R*) 3-hydroxyleucine (note this has the same relative stereochemistry as the (2*S*,3*S*)-stereochemistry of (*L*)-Asn hydroxylation^12,20^), because the *pro*-3*R* hydrogen is closer to the iron (3.4 Å) than the *pro*-3*S* hydrogen (3.9 Å) consistent with the calculations. However, the relative proximity (as observed by crystallography) of the *pro*-3*R* and *pro*-3*S* hydrogens to the iron is “swapped” in the (*D*)-Leu substrate, being 4.1 and 3.2 Å from the iron, respectively (Fig. 6c), in support of the calculations predicting that mono-hydroxylated (*D*)-Leu has the (2*R*,3*S)-*stereochemistry. To test this, we synthesized racemic mixtures of (2*S*,3*R*)/(2*R*,3*S*) and (2*R*,3*R*)/(2*S*,3*S*) 3-hydroxyleucines by established procedures (see Supplementary Methods for details). These were used as standards in amino acid analyses of 3-hydroxyleucine from an ankyrin repeat peptide made synthetically and a peptide product after incubation with FIH (Supplementary Fig. 5). The results from the amino acid analyses imply that (2*R*,3*S*) mono-hydroxylated leucine is produced after incubation of the (*D*)-Leu substrate, in agreement with the computational predictions and crystallography. It is noteworthy that this stereochemical outcome is the opposite to that observed for studied FIH-catalysed C3 hydroxylations with (*2S*)-substrates^12,20^.

## Discussion

Along with the cytochrome 450 and radical SAM enzymes, the 2OG oxygenases form one of the most flexible of all oxidizing enzyme superfamilies^7,10,35^. In animals, the identified reactions catalysed by 2OG oxygenases, are to date substantially limited to single hydroxylations and N-methyl demethylations proceeding *via* hydroxylation (as well as related alcohol and aldehyde oxidations)^2,9^. In plants and microbes, 2OG oxygenases catalyse a much wider range of reactions, including di-hydroxylations of the same residue^2,9^. Thus, in yeasts, a ribosomal protein (RPS23) prolyl hydroxylase catalyses C3 and C4 prolyl hydroxylation, whereas the homologous enzyme in animals apparently catalyses only C3 prolyl hydroxylation^36–38^. There is also evidence for dihydroxylation of lysine residues in diatoms^39^, although the enzyme(s) catalyzing these reactions are unknown. Although its biological/cellular relevance is unclear, if any, the observation that a human 2OG oxygenase, FIH, can catalyse dihydroxylation at different carbons of the same protein residue is thus of interest, because it suggests that the selectivity of human 2OG oxygenases may extend beyond what is presently perceived likely.

In some cases, 2OG oxygenase selectivity is governed by targeting domains in addition to the catalytic domain^40^, whereas in others including FIH, the catalytic domain appears to be, at least principally, responsible for enabling substrate and product selectivity^18,20,41^. Some human 2OG oxygenases acting on proteins appear to be highly selective for specific substrates, whereas others appear much more promiscuous, notably JMJD6 and FIH^12,14,15,21,42,43^. Both of these belong to the JmjC family of 2OG oxygenases, which extends from bacteria to humans^18^. Some JmjC oxygenases catalyse N-methyl demethylations, whereas others catalyse formation of stable alcohol products via unmodified proteinogenic residue side chain oxidations^9,41^. JMJD6 catalyses the (3*S*)-hydroxylation of multiple lysine residues in arginine–serine-rich regions of splicing regulatory proteins and other substrates^43,44^. FIH was originally identified as interacting with HIF^45^, then shown to catalyse C3 hydroxylation of an asparagine-residue in the C-terminal transcriptional activation domain of HIF-α isoforms, a modification that hinders binding of HIF to the CBP/p300 histone acetyl transferases^16–18^. FIH also interacts with, and hydroxylates many ARD proteins, with hydroxylated residues including not only asparagines but also aspartates and histidines, also at the C3 position (and at least with studied polar side chain residues, the reaction proceeds to give the (3*S*)-stereochemistry products, as with HIF-α hydroxylation)^13–15,21^.

Studies on the residue selectivity of isolated FIH using ARD sequences have revealed, albeit at a high enzyme:substrate ratio (as used in our work), that FIH can accept even substrates with hydrophobic side chains and has potential for catalyzing desaturation and hydroxylation^12,15^. These studies were all carried out with (*L*)/(*2S*)-substrates. We are interested in exploring (*D*)/(*2R*)-substrates for 2OG oxygenases, in part because of the increased prevalence of (*D*)-residues in aged cells^46^. Work with JmjC histone demethylases has implied that, at least in some cases, these enzymes are specific for the (*L*)-substrate stereochemistry^47^. By contrast, the results presented here clearly demonstrate that isolated FIH can accept (*D*)-substrates, further expanding the scope of catalysis by isolated FIH.

The substrate/residue selectivity of FIH differs for (*L*)- and (*D*)-substrates, as shown by differences in the rank order of conversion of different C-2 epimeric residues. The product selectivity can differ for the same residue with epimeric C-2 stereochemistry. This was most dramatically illustrated in the case of the epimeric C-2 leucine substrates, which we investigated by both experimental and computational studies. In the case of the (*L*)-leucine substrate, a single hydroxylation is observed, as is typical for FIH^12^. However, with (*D*)-leucine we observed a C3/C4 dihydroxylated product. This work echoes observations on VioC, which is naturally an (*L*)/(*2S*)-arginine 3-hydroxylase, but which in the case of the unnatural (*D*)/(*2R*)-arginine substrate analogue efficiently catalyses an alternative reaction, i.e., oxidative deamintion to give a 2-oxo acid product^48^.

Crystallographic analyses of FIH in complex with various (*D*)-CA peptides implies that the C-2 leucine stereochemistry has a deterministic influence on whether or not hydroxylation occurs (including both first and the second hydroxylations with (*D*)-Leu), due to changes in the side chain binding modes. Using ab initio density functional calculations at the B3LYP/6–31 G** level of theory, we investigated structural and energetic features of the (*L*)- and (*D*)-leucine hydroxylation using a simplified model. The results imply stereoselective formation of (2*S*,3*R*) 3-hydroxyleucine and (2*R*,3*S*) 3-hydroxyleucine from (*L*)- and (*D*)-leucine-containing substrates, respectively, consistent with the experimental observations. The observation that (*D*)-leucine, but not (*L)*-leucine, undergoes two two-electron oxidations to give C3/C4 di-hydroxyleucine appears to reflect increased conformational flexibility in binding of (*D*)-leucine (Fig. 6). Notably, we did not observe significant reaction on incubation of the (2*R*,3*S*) mono-hydroxylated (*D*)-Leu peptide with FIH (Supplementary Figure 1g). The structural and modelling studies suggest it is possible that the C3 alcohol of the mono-hydroxylated (*D*)-Leu product may form a hydrogen bond with the carboxylate of the iron-binding Asp201, thus hindering productive catalysis at the iron-binding site (Fig. 6d); however, this does not explain why we saw more dihydroxylated product from the unhydroxylated (*D*)-Leu substrate. Thus, factors other than those identified here must also be relevant; indeed, although some of the factors determining the catalytic efficiency of FIH with different substrates have been identified (including residue, local sequence context and ARD fold stability), there is little information on the necessary conformational changes required for the canonical ARD structure to bind productively at the FIH active site^13,21–23^.

The results presented here and elsewhere^12–15,21,23^ demonstrate flexibility in FIH catalysis and suggest FIH is a good target for mutagenesis aimed at producing posttranslational protein modifications of choice. Thus, FIH-catalysed hydroxylation or desaturation could, e.g., be used to increase proteolytic thermodynamic stability, induce conformational changes or enable further posttranslational modifications. Finally, the results raise the possibility that oxygenase related (*L*) to (*D*) C-2 residue epimerization could regulate protein oxygenase function, perhaps even in an oxygen/hypoxia-regulated capacity. Posttranslational hydroxylation has not been studied in the context of protein ageing but it has the potential to impact on aging via altering stability or promoting/hindering repair. The prevalence of (*D*)-residues is reported to increase in protiens with aging^46^. Given that FIH differently acts on (*L*)- and (*D*)-residues, it (or other oxygenases acting on proteins) has the potential to be involved in the regulation of protein aging. Given the promiscuity of FIH, there are various ways this could be envisaged to occur. Isoaspartyl residues are formed spontaneously from aspartyl and asparaginyl residues, and are a mark of protein ageing^49,50^. Thus, of particular interest, given the difference in selectivity of FIH for (*L*)- over (*D*)-Asp residues is a role for it in regulating isoaspartyl residue formation/repair.

## Methods

### FIH incubation assays

Small-scale assays used 0–100 μM FIH, 200 μM Fe(II), 50 μM peptide, 2 mM ascorbate and 1 mM 2OG in 100 mM sodium HEPES/150 mM NaCl pH 7.6 buffer^12^. Assays were incubated at 37 °C for 2 h and quenched with 5% aqueous (v/v) formic acid. The reaction mixture was then co-crystallized with an equal quantity of saturated α-cyano-4-hydroxycinnamic acid matrix solution before analysis with MALDI-ToF-MS.

### Reactions with NaIO_4_ and biotin hydrazide

Ten microlitres of NaIO_4_ (5 mM in Milli-Q purified H_2_O) was added to an HPLC-purified solution of dihydroxylated (*D*)-Leu peptide (∼1 mM in Milli-Q purified H_2_O) in a 100 µL PCR tube, and the reaction mixture was left at room temperature for 2 h^28^. Biotin hydrazide (1 mM in Milli-Q purified H_2_O) was added, and the reaction mixture was incubated at 37 °C for 2 h before analysis by MALDI-TOF-MS.

### MALDI-ToF-MS

One microlitre of saturated α-cyano-4-hydroxycinnamic acid matrix was mixed with 1 µL reaction mixture on a sample plate. The plate was air-dried prior to analysis on a Waters MALDI micro MX^TM^ mass spectrometer in the positive ion reflectron mode. Parameters: Laser energy: 175–250 V, Pulse: 2050 V, Detector: 2600 V, Suppression: 125. Data were analysed using MassLynx 4.0 and spectra obtained were background subtracted (polynomial order = 16, % below curve = 10, tolerance = 0.01).

### Amino acid analysis

Amino acid analyses were carried out using the acid hydrolysis method as described in Mantri et al.^44^.

### Computational, crystallographic and synthetic methods

See Supplementary Methods.

## Supplementary information

Supplementary Information

## Data Availability

The main data supporting the findings of this study are available within the paper and its Supplementary Information file. The PDB accession codes for novel crystal structures are 4JAA, 6RUJ and 4NR1. Other relevant data are available from the corresponding author upon reasonable request.
