# Supplementary material for: A human protein hydroxylase that accepts D-residues
Source: Commun Chem. 2020 May 1;3:52. doi: 10.1038/s42004-020-0290-5 (PMC9814778; doi:10.1038/s42004-020-0290-5)
Supplement: Supplementary file 1 — Supplementary Information [file 42004_2020_290_MOESM1_ESM.pdf]

Supplementary Information

A Human Protein Hydroxylase that Accepts *D*-Residues

Hwanho Choi[a], Adam P. Hardy[a], Thomas M. Leissing[a], Rasheduzzaman Chowdhury[a], Yu Nakashima[a], Wei Ge[a], Marios Markoulides[a], John S. Scotti[a], Philip A. Gerken[a], Helen Thorbjornsrud[a], Dahye Kang[b,c], Sungwoo Hong[b,c], Joongoo Lee[d], Michael A. McDonough[a], Hwangseo Park[*e] and Christopher J. Schofield[*a]

[a]Department of Chemistry, Chemistry Research Laboratory, University of Oxford, Mansfield Road, Oxford, OX1 3TA, United Kingdom.

[b]Center for Catalytic Hydrocarbon Functionalizations, Institute for Basic Science (IBS), Daejeon, 34141 Korea.

[c]Department of Chemistry, Korea Advanced Institute of Science and Technology (KAIST), Daejeon, 34141 Korea.

[d]Department of Chemical and Biological Engineering, Northwestern University, 2145 Sheridan Rd, Evanston, IL 60208, United States of America.

[e]Department of Bioscience and Biotechnology, Sejong University, 209 Neungdong-ro, Kwangjin-gu, Seoul 05006, Korea.

* Correspondence to: christopher.schofield@chem.ox.ac.uk and hspark@sejong.ac.kr

Supplementary Figure 1. MALDI-MS analysis of FIH assays. FIH catalyzes oxidation of (*D*)-residues in a consensus ankyrin repeat derived sequence with (a) acidic; (b) amide; (c) basic; (d) nucleophilic; (e) aromatic, (f) hydrophobic side chains and (g) non-proteinogenic amino acids at the potential hydroxylation position ((*D*) / (*2R*) = d). Peptides were incubated in the absence (bottom panels) or presence (top panels) of FIH under conditions described in the Methods. Modification of peptides after incubation with FIH is indicated. Assays were carried out at least in duplicate, with representative spectra shown.

a

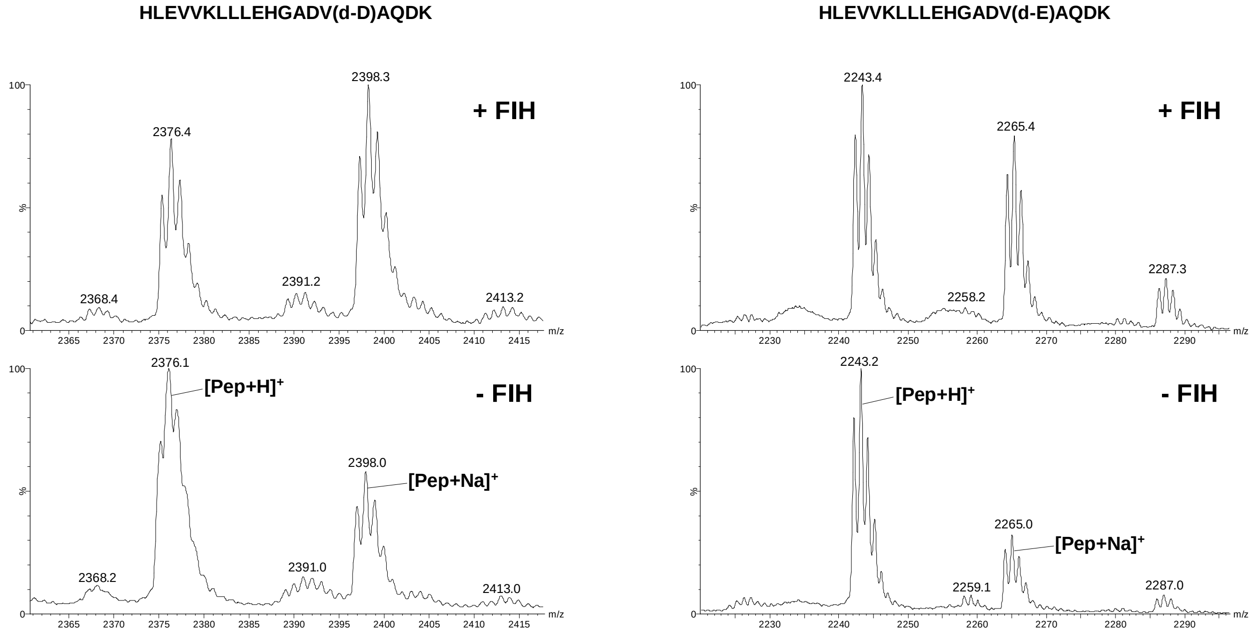

b

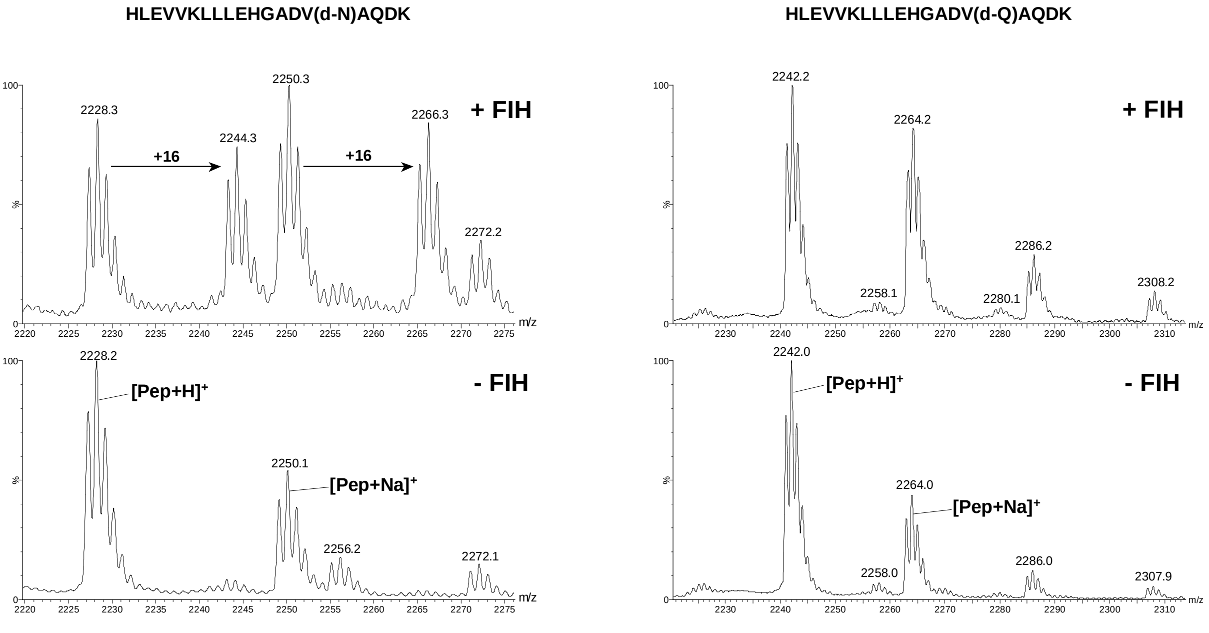

c

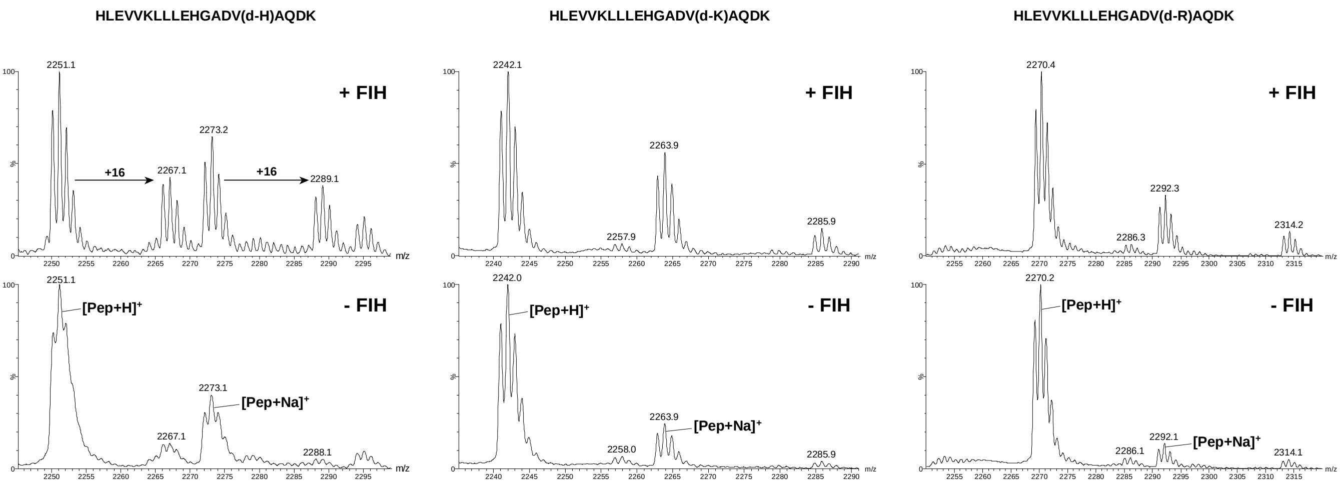
HLEVVKLLLEHGADV(d-H)AQDK
HLEVVKLLLEHGADV(d-K)AQDK
HLEVVKLLLEHGADV(d-R)AQDK
+ FIH
- FIH
+16
[Pep+H]+
[Pep+Na]+
2251.1
2267.1
2273.2
2289.1
2273.1
2288.1
2242.1
2257.9
2263.9
2285.9
2242.0
2258.0
2270.4
2286.3
2292.3
2314.2
2270.2
2286.1
2292.1
2314.1
m/z

d

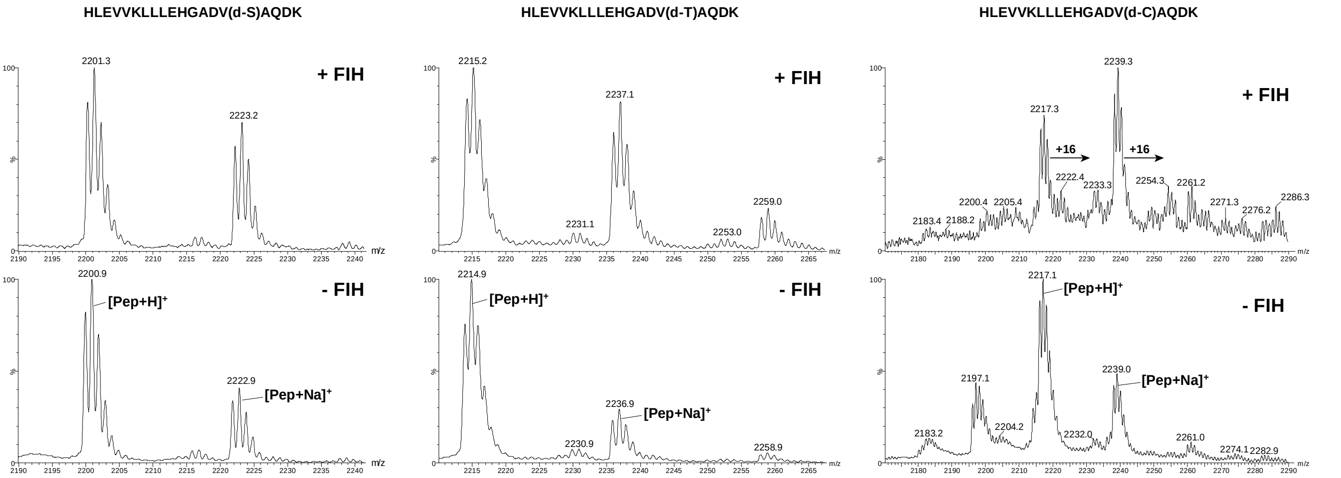
HLEVVKLLLEHGADV(d-S)AQDK
HLEVVKLLLEHGADV(d-T)AQDK
HLEVVKLLLEHGADV(d-C)AQDK
+ FIH
- FIH
+16
[Pep+H]+
[Pep+Na]+
2201.3
2223.2
2200.9
2222.9
2215.2
2231.1
2237.1
2253.0
2259.0
2214.9
2230.9
2236.9
2258.9
2239.3
2217.3
2183.4
2188.2
2200.4
2205.4
2222.4
2233.3
2254.3
2261.2
2271.3
2276.2
2286.3
2217.1
2197.1
2183.2
2204.2
2232.0
2239.0
2261.0
2274.1
2282.9
m/z

e

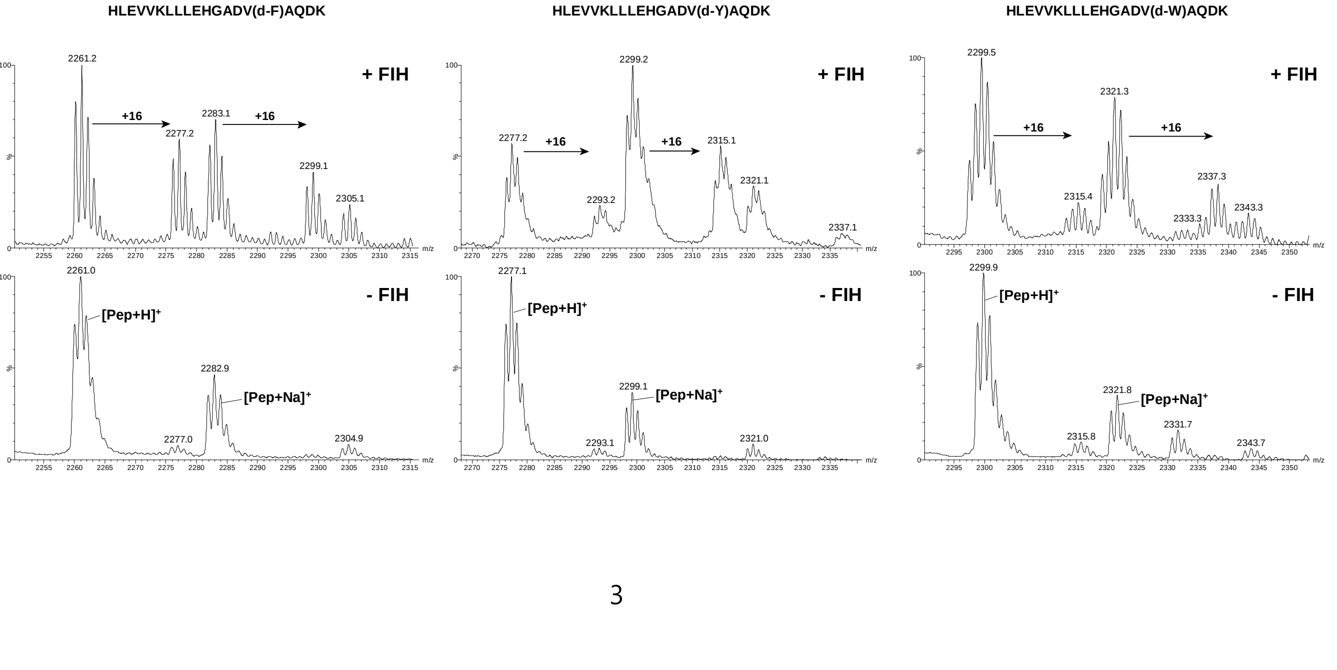
HLEVVKLLLEHGADV(d-F)AQDK
HLEVVKLLLEHGADV(d-Y)AQDK
HLEVVKLLLEHGADV(d-W)AQDK
+ FIH
- FIH
+16
[Pep+H]+
[Pep+Na]+
2261.2
2277.2
2283.1
2299.1
2305.1
2261.0
2277.0
2282.9
2304.9
2277.2
2293.2
2299.2
2315.1
2321.1
2337.1
2277.1
2293.1
2299.1
2321.0
2299.5
2315.4
2321.3
2333.3
2337.3
2343.3
2299.9
2315.8
2321.8
2331.7
2343.7
m/z

f

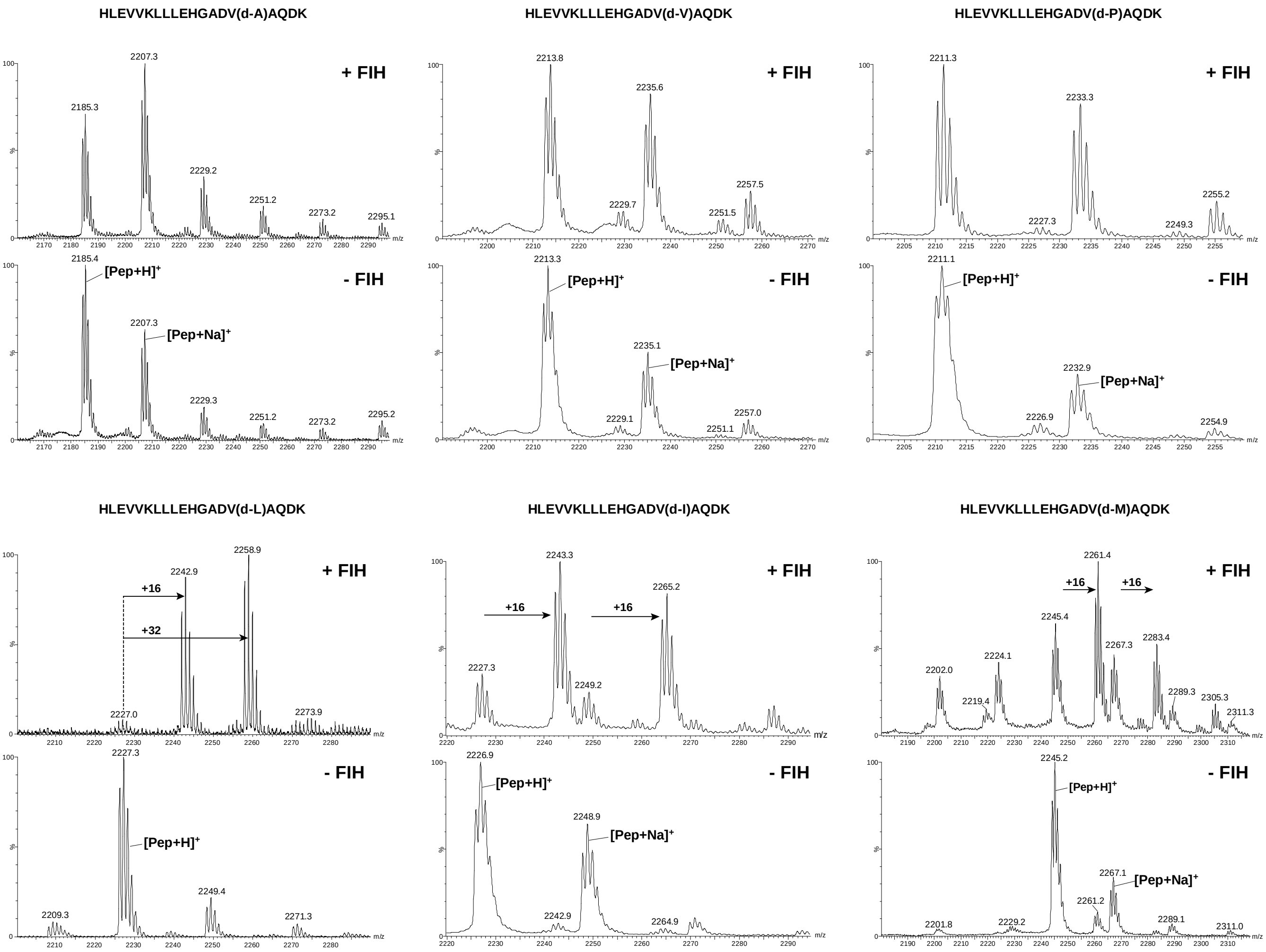
HLEVVKLLLEHGADV(d-A)AQDK
+ FIH
- FIH
[Pep+H]+
[Pep+Na]+
2185.3
2207.3
2229.2
2251.2
2273.2
2295.1
2185.4
2207.3
2229.3
2251.2
2273.2
2295.2
HLEVVKLLLEHGADV(d-V)AQDK
+ FIH
- FIH
[Pep+H]+
[Pep+Na]+
2213.8
2235.6
2229.7
2257.5
2251.5
2213.3
2235.1
2229.1
2257.0
2251.1
HLEVVKLLLEHGADV(d-P)AQDK
+ FIH
- FIH
[Pep+H]+
[Pep+Na]+
2211.3
2233.3
2227.3
2255.2
2249.3
2211.1
2232.9
2226.9
2254.9
HLEVVKLLLEHGADV(d-L)AQDK
+ FIH
- FIH
+16
+32
[Pep+H]+
2258.9
2242.9
2227.0
2273.9
2227.3
2209.3
2249.4
2271.3
HLEVVKLLLEHGADV(d-I)AQDK
+ FIH
- FIH
+16
+16
[Pep+H]+
[Pep+Na]+
2243.3
2265.2
2227.3
2249.2
2226.9
2248.9
2242.9
2264.9
HLEVVKLLLEHGADV(d-M)AQDK
+ FIH
- FIH
+16
+16
[Pep+H]+
[Pep+Na]+
2261.4
2245.4
2267.3
2283.4
2224.1
2202.0
2219.4
2289.3
2305.3
2311.3
2245.2
2267.1
2261.2
2201.8
2229.2
2289.1
2311.0
m/z

g

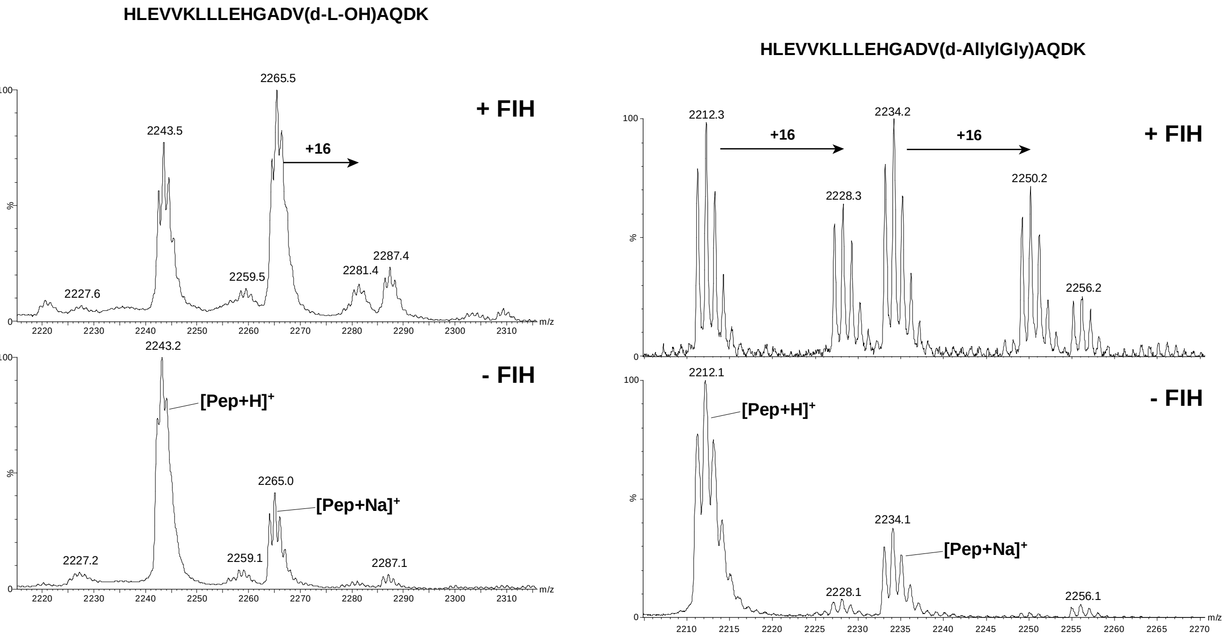
HLEVVKLLLEHGADV(d-L-OH)AQDK
+ FIH
- FIH
+16
[Pep+H]+
[Pep+Na]+
2265.5
2243.5
2227.6
2259.5
2281.4
2287.4
2243.2
2265.0
2227.2
2259.1
2287.1
HLEVVKLLLEHGADV(d-AllylGly)AQDK
+ FIH
- FIH
+16
+16
[Pep+H]+
[Pep+Na]+
2212.3
2234.2
2228.3
2250.2
2256.2
2212.1
2234.1
2228.1
2256.1
m/z

Supplementary Table 1. FIH catalyzes the oxidation of multiple (*D*)-residues in consensus ankyrin sequences (CA_X). (a) Results from incubation of consensus ankyrin peptides (with the potentially hydroxylated residue in bold. Masses were obtained from MALDI-MS. Note that in the case of the (*D*)-Leu substrate, two products were observed. (b) Extent of hydroxylation of ankyrin repeat peptides containing (*D*)-residues in the potential hydroxylation position. Note (*D*)-Met is not included because incubation with FIH appeared to produce several products. % Hydroxylation for (*D*)-Leu includes both mono- and di-hydroxylation. Studies on the analogous (*L*)-residues in the CA sequence have been previously reported,[1] with the following order of efficiency: CA_N >> CA_H ≈ CA_S ≈ CA_D >> CA_I ≈ CA_L.

a

Consensus Ankyrin Repeat Peptide	Name	Substrate mass (Da)	Product mass(es) (Da)
HLEVVKLLLEHGADV(**D-N**)AQDK	CA_N	2228.5	2244.3 (+16 Da)
HLEVVKLLLEHGADV(**D-D**)AQDK	CA_D	2229.5	No Product Detected
HLEVVKLLLEHGADVD(**D-H**)AQDK	CA_H	2251.6	2267.1 (+16 Da)
HLEVVKLLLEHGADV(**D-S)**AQDK	CA_S	2201.5	No Product Detected
HLEVVKLLLEHGADV(**D-L**)AQDK	CA_L	2227.6	2243.6 (+16 Da) ; 2259.6 (+32 Da)
HLEVVKLLLEHGADV(**D-I**)AQDK	CA_I	2227.6	2243.3 (+16 Da)
HLEVVKLLLEHGADV(**D-W**)AQDK	CA_W	2300.6	2321.3 (+16 Da)
HLEVVKLLLEHGADV(**D-T**)AQDK	CA_T	2215.5	No Product Detected
HLEVVKLLLEHGADV(**D-K**)AQDK	CA_K	2242.6	No Product Detected
HLEVVKLLLEHGADV(**D-F**)AQDK	CA_F	2261.6	2277.2 (+16 Da)
HLEVVKLLLEHGADV(**D-P**)AQDK	CA_P	2211.5	No Product Detected
HLEVVKLLLEHGADV(**D-A**)AQDK	CA_A	2185.5	No Product Detected
HLEVVKLLLEHGADV(**D-M**)AQDK	CA_M	2245.6	Multiple Oxidations (non-enzymatic)
HLEVVKLLLEHGADV(**D-Y**)AQDK	CA_Y	2277.6	2299.2 (+16 Da)
HLEVVKLLLEHGADV(**D-V**)AQDK	CA_V	2213.5	No Product Detected
HLEVVKLLLEHGADV(**D-R**)AQDK	CA_R	2270.6	No Product Detected
HLEVVKLLLEHGADV(**D-Q**)AQDK	CA_Q	2242.5	No Product Detected
HLEVVKLLLEHGADV(**D-C**)AQDK	CA_C	2217.6	2233.3 (+16 Da)
HLEVVKLLLEHGADV(**D-E**)AQDK	CA_E	2243.5	No Product Detected
HLEVVKLLLEHGADV(**D-allylglycine**)AQDK	CA_Allyl	2211.6	2227.6 (+16 Da)
HLEVVKLLLEHGADV(**D-L-OH**)AQDK	CA_L-OH	2243.6	Small amount 2259.6 (+16 Da)

b

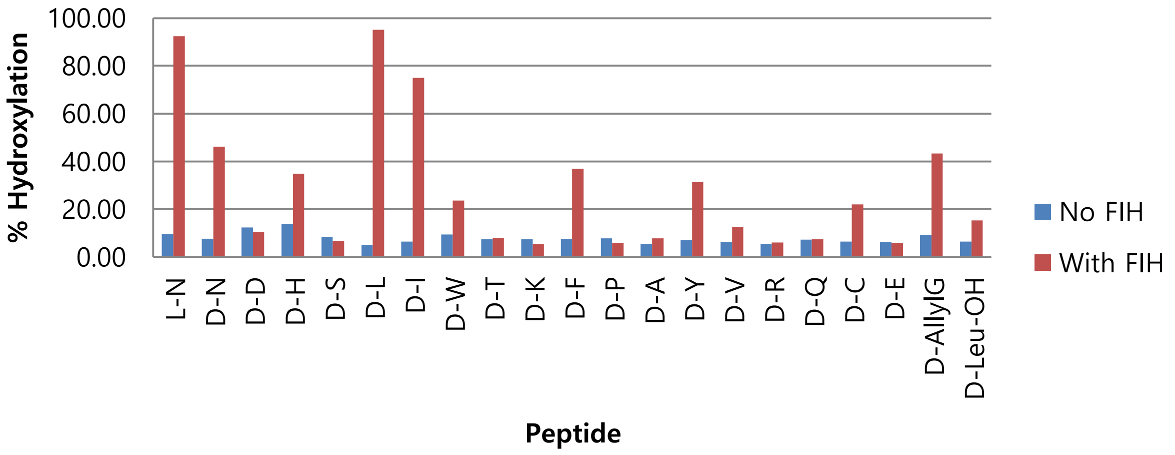
% Hydroxylation
100.00
80.00
60.00
40.00
20.00
0.00
L-N
D-N
D-D
D-H
D-S
D-L
D-I
D-W
D-T
D-K
D-F
D-P
D-A
D-Y
D-V
D-R
D-Q
D-C
D-E
D-AllylG
D-Leu-OH
Peptide
No FIH
With FIH

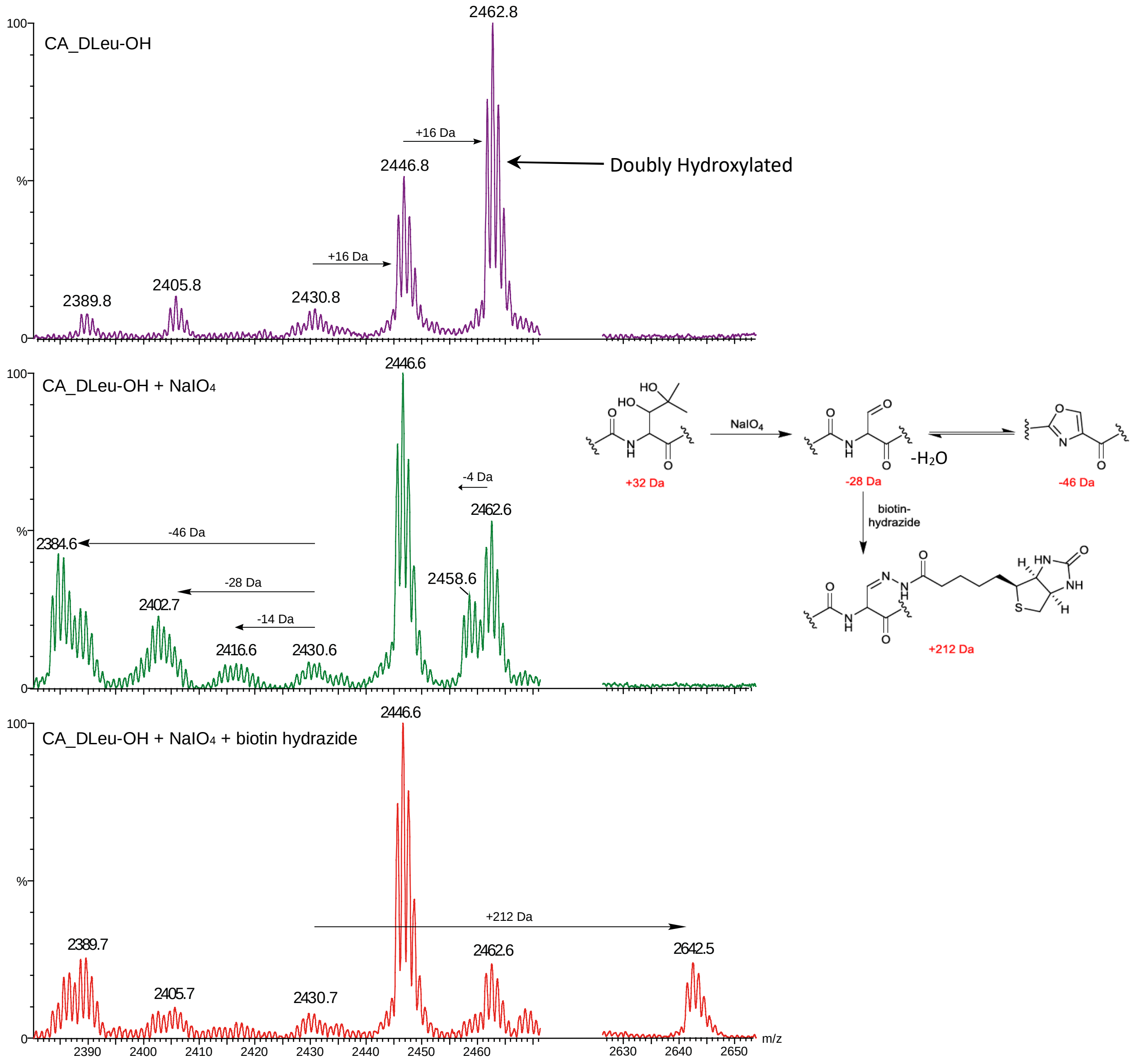

Supplementary Figure 2. Evidence for the presence of two hydroxylations (+32 Da) with the (*D*)-Leu peptide HLEVVKLLLEHGADV(*D*-L)AQDKFG. (a) MALDI-MS after FIH treatment; (b) after treatment of the product in (a) with $NaIO_4$ (showing proposed products, with mass increments relative to the unmodified peptide mass); (c) treatment of the product in (b) with biotin hydrazide.

Supplementary Methods

Computational Methods

The starting coordinates for *ab initio* DFT calculations were extracted from a crystal structure of FIH complexed with N-oxalylglycine (NOG, a close 2OG analogue) and peptides including (*D*)- or (*L*)-leucine residues (PDB entry: 4JAA or 4B7E respectively). To construct a reaction model for the calculations, the substrate was simplified to a (*D*)- or (*L*)-leucine (*i.e.* with an N-methylated amino and C-terminal methyl ketone, hereafter referred to as (*D*)-Leu or (*L*)-Leu); NOG was modified to 2OG. Methylimidazole and an acetate ion were used to mimic the His- and Asp- side-chains coordinating to the iron. The vacant coordination site was occupied by the proposed reactive oxygen species (ferryl intermediate) to give the model in Supplementary Figure 3, which is based on the FIH mechanism reviewed in Chowdhury *et al.*[2] and involves reaction of dioxygen with the metal coordinating ketone of 2OG, leading to the reactive Fe(IV)=O species responsible for hydroxylation.

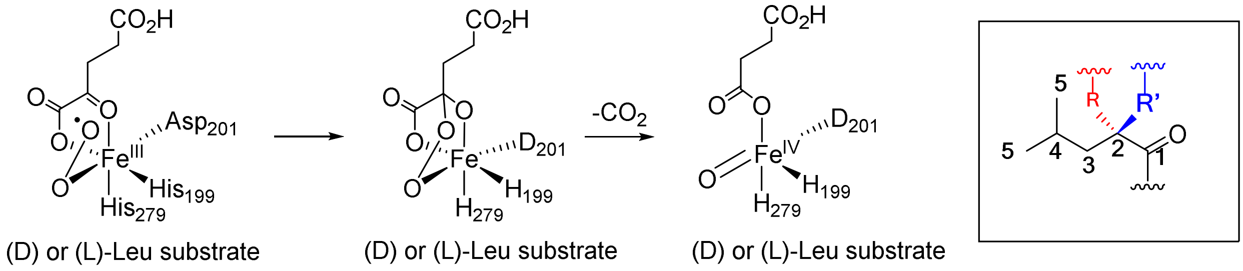

Supplementary Figure 3. Scheme showing selected steps during formation of the catalytically active form of FIH. The numberings of the carbon atoms of leucine used are indicated.

Geometries corresponding to the energy minima on the reaction path were optimized using Gaussian09. The geometry optimizations were carried out with the 6-31G** basis set for all atoms. To calculate the electronic energies of the reactants and products in complex with the FIH active site, we used the B3LYP hybrid density functional. This consists of Becke's hybrid gradient-corrected exchange functional[3] and the gradient-corrected correlation functional of Lee, Yang, and Parr.[4] The spin multiplicity of the iron ion was assumed to be sextet. Based on the reaction pathway optimized structures, we investigated mechanistic aspects of the hydroxylation of (*D*)- and (*L*)-leucine-residues. The nature of each stationary-state structure encountered on the reaction pathways was investigated by the number of imaginary frequencies obtained by diagonalization of the analytical hessian matrix. Each transition state was characterized by a single negative eigenvalue; the corresponding imaginary vibrational frequency was related to the motion that would connect the expected starting and final minima.

Crystallography

Supplementary Table 2. Crystallographic data and refinement statistics.

Protein: Substrates	FIH:CAP(*D*-leucine)	FIH:CAP((2*R*,3*S*) 3-hydroxyleucine)	FIH:CAP(*D*-allylglycine)
X-ray source	Diamond Light Source beamline I04-1	Diamond Light Source beamline I04	Diamond Light Source beamline I04-1
Wavelength (Å)	0.9173	0.97942	0.9173
PDB Acquisition Code	4JAA	6RUJ	4NR1
Resolution (Å)	2.39 (2.55–2.39)[§]	2.42 (2.46-2.42)[§]	2.68 (2.75–2.68)[§]
Space group	$P\ 4_1\ 2_1\ 2$	$P\ 4_1\ 2_1\ 2$	$P\ 4_1\ 2_1\ 2$
Unit Cell Dimensions (a Å, b Å, c Å)	86.08, 86.08, 147.03	86.40, 86.40, 147.26	86.37, 86.37, 148.72
Molecules per a.u.	1	1	1
Total Number of Reflections Observed	112791	281827	164722
Number of Unique Reflections	22451 (3937)[§]	22013 (1051)[§]	16485 (1178)[§]
Redundancy	5.0 (4.6)[§]	12.8 (13.4)[§]	10.0 (10.8)[§]
Completeness (%)	99.3 (98.7)[§]	100.0 (98.2)[§]	99.9 (99.9)[§]
Wilson B	44.6	60.5	67
I/σ(I)	9.3 (2.6)[§]	10.6 (1.0)[§]	17.3 (2.9)[§]
[§§]R_{merge}	0.089	0.148	0.1
*R_{cryst}	0.184	0.209	0.164
[†]R_{free}	0.219	0.257	0.21
¶RMS deviation	0.01 (1.1°)	0.01 (1.2°)	0.01 (1.2°)
Average B factors (Å^2)	41.7	81.1	63.4
Number of Water Molecules	120	93	54

[§]Parentheses indicate the highest resolution shell.

[§§]$R_{merge} = \sum_j \sum_h \left| I_{hj} - < I_h > \right| / \sum_j \sum_h \left| < I_h > \right| \times 100$

*$R_{cryst} = \sum \left\| F_{obs} \right| - \left| F_{calc} \right\| / \left| F_{obs} \right| \times 100$

[†]R_{free}, based on 2-5% of the total reflections.

¶RMS deviation from ideality for bonds (followed by the value for angle).

Crystals of FIH in complex with Zn(II), NOG and consensus ankyrin repeat peptides (CAPs) were grown in sitting drops using the vapour diffusion method (drop size: 200-300 nL) at 293 K in 96-well Intelliplates (Art Robbins). Crystals were cryo-protected by transfer to 25% (v/v) glycerol in the well solution and then harvested using nylon loops (Hampton Research) and cryo-cooled by plunging in liquid nitrogen. Data were collected at 100 K using single crystals at Diamond Light Source beamline I04-1 [FIH:CAP((*D*)-leucine) and FIH:CAP((*D*)-allylglycine)] with a

MarMosaic 300 mm CCD detector, and Diamond Light Source beamline I04 [FIH:CAP((2*R*,3*S*) 3-hydroxyleucine)] with a Eiger2 X 16M detector. Data were then indexed, integrated, and scaled using SCALA [FIH:CAP((*D*)-leucine) and FIH:CAP((*D*)-allylglycine)][5] and xia2 [FIH:CAP((2*R*,3*S*) 3-hydroxyleucine)].[6] Structures were determined by molecular replacement (MR) using the MR-PHASER[7] subroutine of PHENIX[8] using *H. sapiens* FIH (PDB ID: 1H2K)[9] as the search model. Model building and refinement were performed iteratively using COOT[10] and PHENIX until converging R and R_{free} no longer decreased. Mn(II), NOG, and CAP were modelled in the final stages of refinement based on the $F_{obs} – F_{calc}$ electron density map.

The overall structures of FIH in complex with (*D*)-leucine, (2*R*,3*S*) 3-hydroxyleucine, and (*D*)-allylglycine CAP reveal that in each case the substrate main chain atoms bind to FIH in a similar overall conformation, consistent with those reported for other similar length FIH substrates (Supplementary Figure 4). Importantly, in the case of the FIH:CAP (2*R*,3*S*) 3-hydroxyleucine structure, the 2.4 Å resolution dataset was sufficient to clearly view the β-hydroxyl group of (*D*)-leucine peptide within the electron density map (Supplementary Figure 4B).

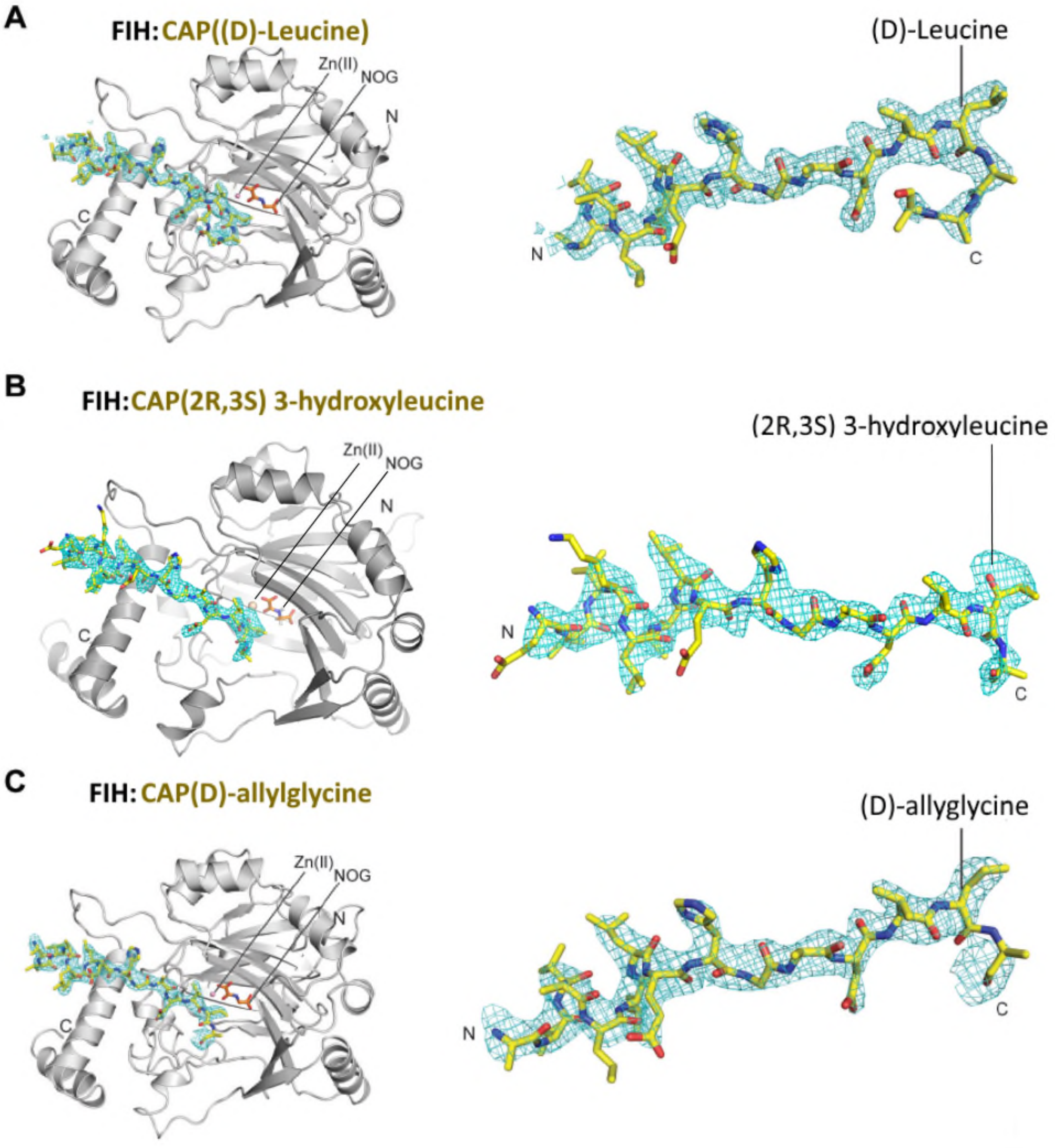

Supplementary Figure 4. Views from crystal structures of FIH in complex with consensus ankyrin (CA) repeat peptides containing (*D*)-residues at the potential hydroxylation position. Views from the overall structure of FIH (only one monomer of the dimer is shown) in complex with: (**A**) CA with (*D*)-leucine (CA N), (**B**) CA with (2*R* ,3*S*) 3-hydroxyleucine, and (**C**) CA with (*D*)-allyglycine); the electron density OMIT $|mF_o - DF_c|$ (shown in cyan mesh) maps are contoured to 3.0 σ. All structures were obtained with Zn(II) substituting for Fe(II) and NOG substituting for 2OG.

Synthesis of (2*R*,3*S*) 3-hydroxyleucine[11-13]

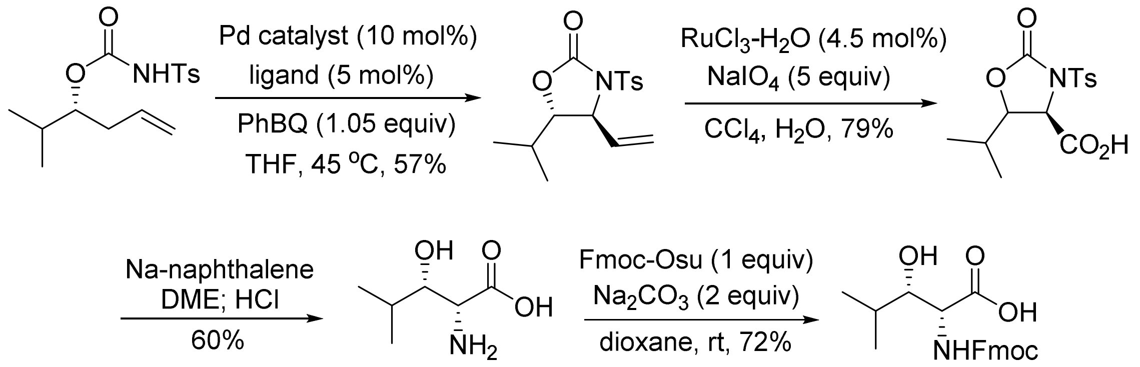

Preparation of (2*R*,3*S*) 3-hydroxyleucine. Reported procedures, including established solid phase peptide synthesis procedures for candidate FIH substrates were used.[1,11-14]

(2*R*,3*S*)-2-((((9H-Fluoren-9-yl)methoxy)carbonyl)amino)-3-hydroxy-4-methylpentanoic acid. To a 100mL round-bottom flask was added 3-hydroxyleucine (560 mg, 3.80 mmol), 1,4-dioxane (15 mL), and Na_2CO_3 0.5 M (15 mL, 7.6 mmol) followed by Fmoc-Osu (1.28, 3.80 mmol). The reaction mixture was stirred at room temperature for 24 h, then poured into Et_2O (15 mL); the aqueous layer was extracted 3 times with Et_2O (15 mL). The aqueous layer was cooled to 0 °C and acidified with 1M HCl to pH to 2-3. The aqueous layer was extracted with AcOEt (40 mL) 3 times. The combined organic layers were washed with brine, dried over Na_2SO_4, filtered, then concentrated under reduced pressure. The residue was purified by flash chromatography hexane/AcOEt, 3:1 then 1:1) to afford the product (1.01 g, 72% yield). $[\alpha]_D^{25}$ = +5.25 (c 1.00, $CHCl_3$), ^{1}H NMR (400 MHz, Methanol-d_4) δ 7.80 (d, J = 7.5 Hz, 2H), 7.68 (t, J = 8.0 Hz, 2H), 7.40 (t, J = 7.5 Hz, 2H), 7.32 (t, J = 7.5 Hz, 2H), 4.46 – 4.36 (m, 3H), 4.24 (t, J = 7.0 Hz, 1H), 3.70 (dd, J = 9.0, 2.5 Hz, 1H), 1.77 – 1.61 (m, 1H), 1.04 (d, J = 6.5 Hz, 3H), 0.93 (d, J = 6.5 Hz, 3H). ^{13}C NMR (101 MHz, Methanol-d_4) δ 157.40, 143.96, 143.66, 141.20, 141.18, 127.37, 126.76, 126.74, 124.83, 119.52, 77.04, 66.60, 47.05, 30.95, 18.23, 18.11. HRMS (ESI$^+$) m/z $[M+Na]^+$. Calculated for $C_{21}H_{23}NNaO_5$: 392.1474, found: 392.1468.

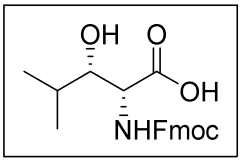

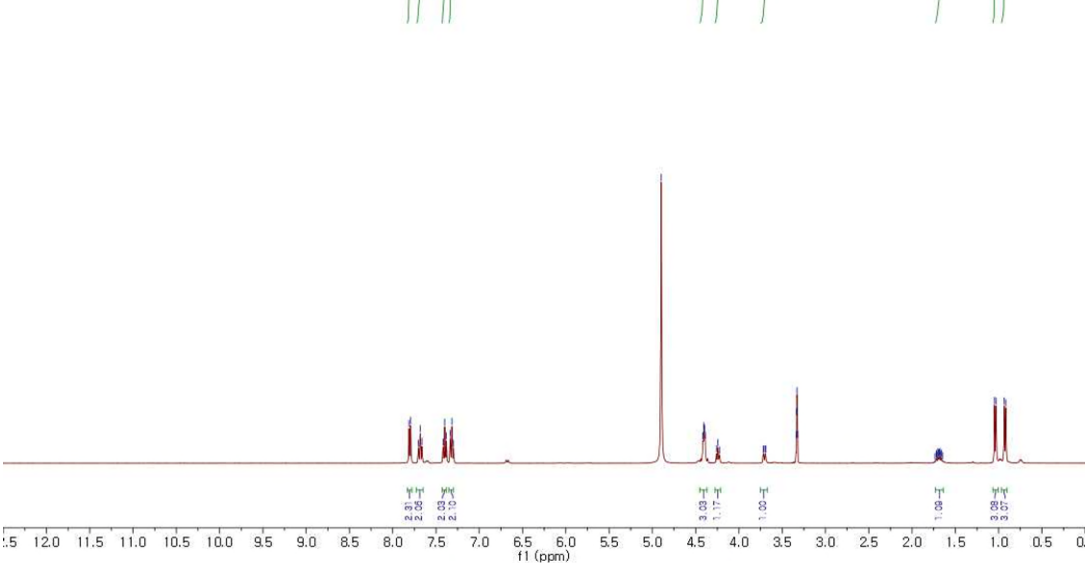

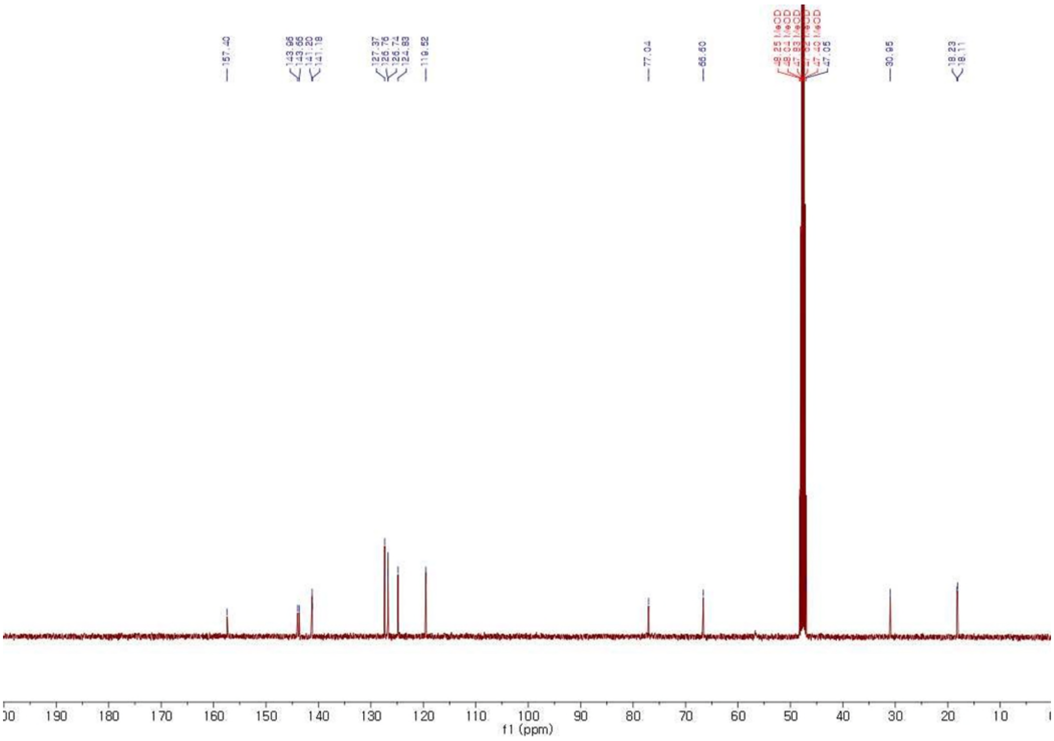

NMR of (2*R*,3*S*)-2-((((9H-Fluoren-9-yl)methoxy)carbonyl)amino)-3-hydroxy-4-methylpentanoic acid. $[\alpha]_D^{25}$ = +5.25 (c 1.00, $CHCl_3$), ^1H NMR (400 MHz, methanol-d_4) δ 7.80 (d, *J* = 7.6 Hz, 2H), 7.68 (t, *J* = 7.8 Hz, 2H), 7.40 (t, *J* = 7.5 Hz, 2H), 7.32 (t, *J* = 7.4 Hz, 2H), 4.46 – 4.36 (m, 3H), 4.24 (t, *J* = 6.9 Hz, 1H), 3.70 (dd, *J* = 9.1, 2.4 Hz, 1H), 1.77 – 1.61 (m, 1H), 1.04 (d, *J* = 6.6 Hz, 3H), 0.93 (d, *J* = 6.7 Hz, 3H). ^{13}C NMR (101 MHz, methanol-d_4) δ 157.40, 143.96, 143.66, 141.20, 141.18, 127.37, 126.76, 126.74, 124.83, 119.52, 77.04, 66.60, 47.05, 30.95, 18.23, 18.11.

Synthesis of racemic 3-hydroxyleucines (5a and 5b)[11]

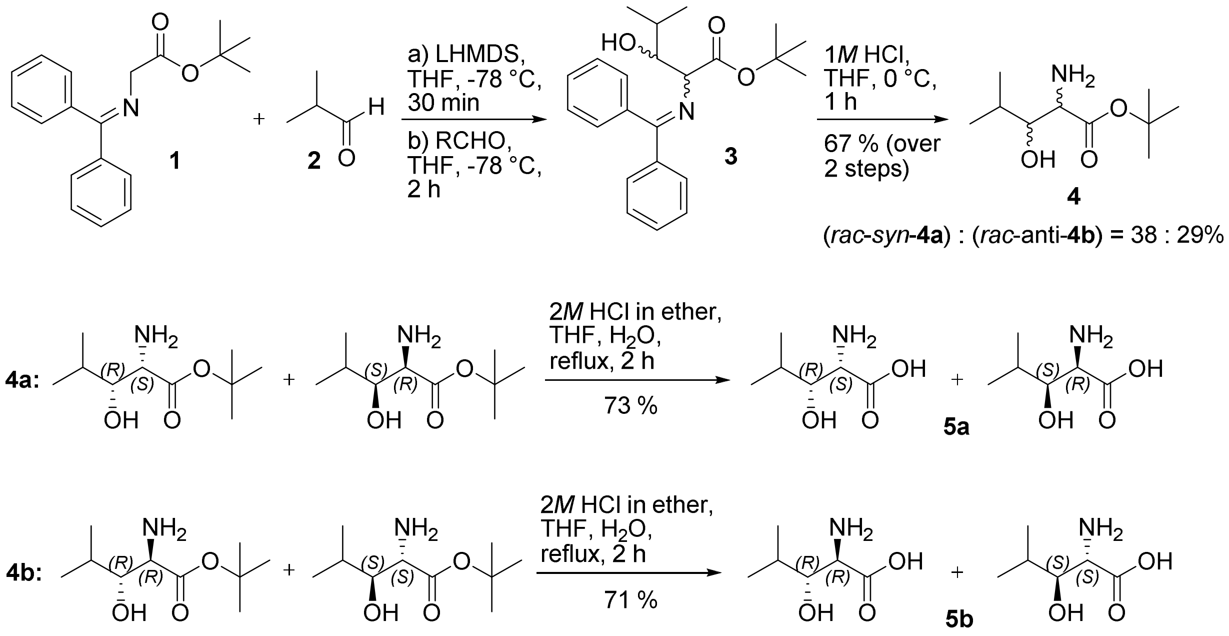

Synthesis of 3-hydroxyleucines **5a** and **5b** followed the procedure of Gomes *et al.*[11] The racemic mixtures of diastereoisomers **(4a)** / **(4b)** were separated by flash chromatography (EtOAc/MeOH = 19:1; **4a**:**4b** = 38:29) for analytical purposes, then hydrolysed to give the corresponding racemic mixtures of 3-hydroxyleucines **5a** and **5b**,[11] respectively. Spectral data for **4a**,[11] **4b**,[11] **5a**[15-17] and **5b**[15-17] were in agreement with those reported.

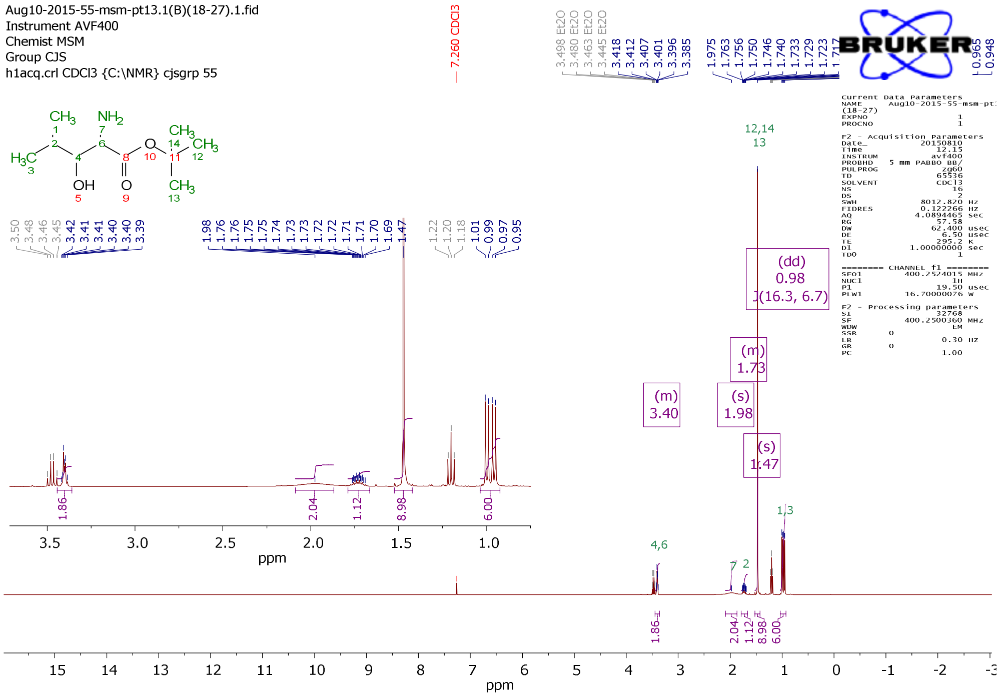

NMR of (2*R*,3*S*/2*S*,3*R*) 3-hydroxyleucine *tert* butyl ester (4a): δ_H (400 MHz, $CDCl_3$) 3.44–3.36 (2H, m, H-4, H-6), 1.98 (2H, br s), 1.79–1.66 (1H, m, H-2), 1.47 (9H, s, H-12, H-13, H-14), 0.98 (6H, dd, *J* = 16.3, 6.7 Hz, H-1, H-3).

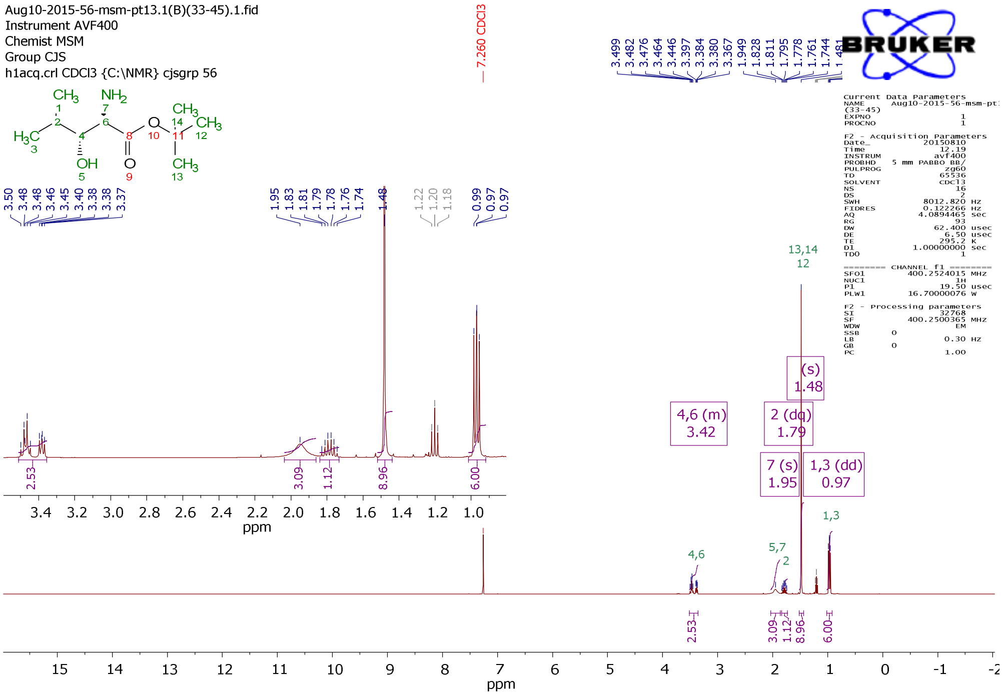

NMR of (2*R*,3*R*/2*S*,3*S*) 3-hydroxyleucine *tert* butyl ester (4b): ***δ*$_H$** (400 MHz, $CDCl_3$) 3.51–3.36 (2H, m, H-4, H-6), 1.95 (3H, br s, NH_2, OH), 1.79 (1H, dq, *J* = 13.5, 6.7 Hz, H-2), 1.48 (9H, s, H-12, H-13, H-14), 0.97 (6 , dd, *J* = 6.8, 5.6 Hz, H-1, H-3).

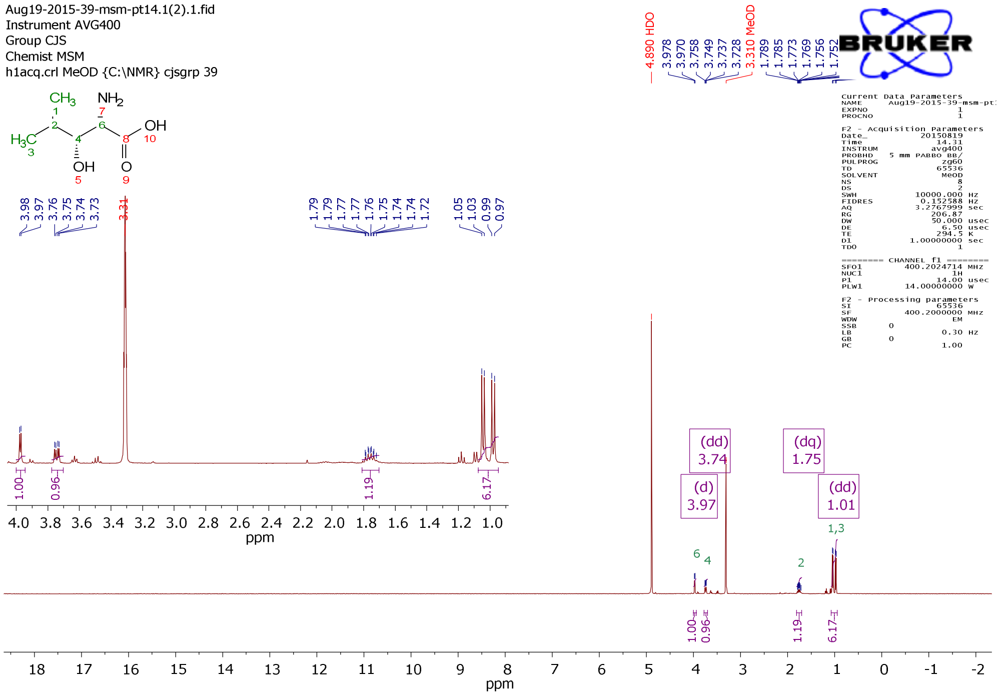

NMR of (2*R*,3*S*/2*S*,3*R*) 3-hydroxyleucine (5a): ***δ*$_H$** (400 MHz, MeOD) 3.97 (1H, d, *J* = 3.4 Hz, H-6), 3.74 (1H, dd, *J* = 8.3, 3.4 Hz, H-4), 1.75 (1H, dq, *J* = 8.2, 6.6 Hz, H-2), 1.01 (6H, dd, *J* = 6.7, 6.7 Hz, H-1,H-3).

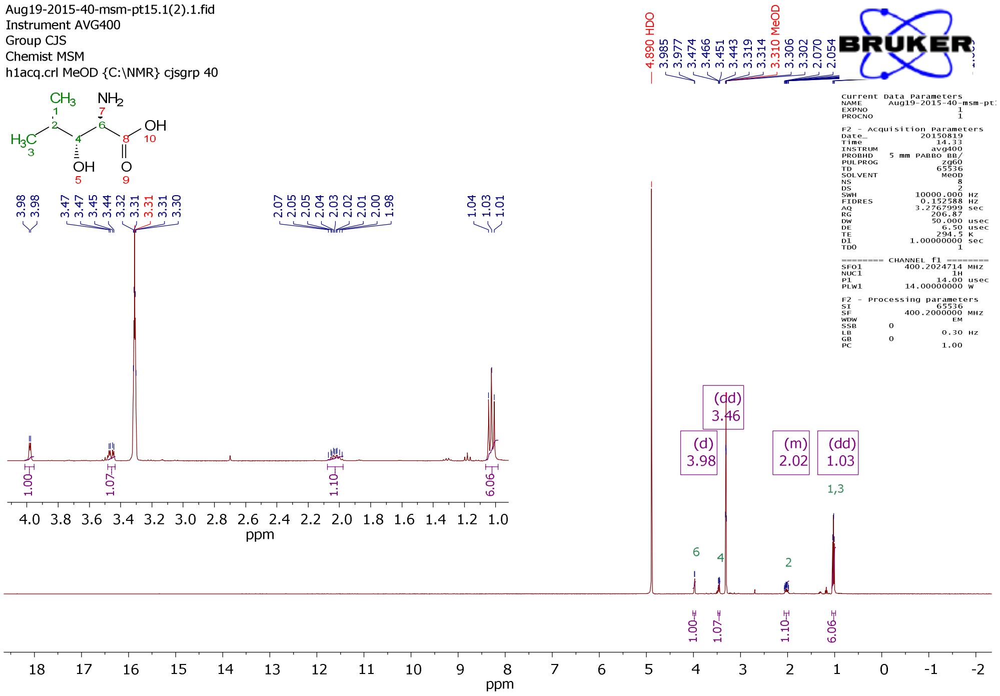

NMR of (2*R*,3*R*/2*S*,3*S*) 3-hydroxyleucine (5b): δ_H (400 MHz, MeOD) 3.98 (1H, d, J = 3.1 Hz, H-6), 3.46 (1H, dd, J = 9.0, 3.2 Hz, H-4), 2.08–1.98 (1H, m, H-2), 1.03 (6H, m, H-1, H-3).

Synthesis of the (2*R*,3*S*) 3-hydroxyleucine containing CA peptide

Peptides were synthesized by reported standard procedures.[1] 2-(1H-Benzotriazole-1-yl)-1,1,3,3-tetramethyluronium hexafluorophosphate (HBTU) and N^{α}-Fmoc protected amino acids were from Novabiochem. Hydroxybenzotriazole (HOBt), N-methylpyrrolidone (NMP), N, N-diisopropylamine (DIEA), N,N-dimethylformamide (DMF), dichloromethane (DCM, anhydrous), diethyl ether, acetonitrile (HP LC grade), guanidine hydrochloride (Gu.HCl), trifluoroacetic acid (TFA), and triisopropylsilane (TIS) were from Sigma-Aldrich. Except for Fmoc-D-β-(2*R*,3*S*)-hydroxyleucine, which was synthesized, all the amino acids for Fmoc-SPPS were from Novabiochem.

Automated synthesis was performed on a 0.25 mmol scale using a peptide synthesizer (Liberty, CEM). Standard chain elongation was performed on Wang resin (Sigma-Aldrich). For each coupling reaction, 0.5 M (1.2 mmol) HBTU, 0.5 M (1.2 mmol) HOBt and 0.2 M Fmoc-Xaa (1.2 mmol) in DMF were added to the resin. Subsequently, 2.0 M DIEA in NMP was added, and the coupling reaction was carried out in a microwave chamber. The Fmoc protecting group was then removed by treatment with 20% (v/v) piperidine in DMF. Side-chain protection was as follows: Fmoc-Glu(OtBu)-OH, Fmoc-Lys(Boc)-OH, Fmoc-His(Trt)-OH, Fmoc-Asp(OtBu)-OH, and Fmoc-Gln(Trt)-OH.

Peptide purification was carried out using a Dionex UltiMate 3000 HPLC instrument. Peptides were dissolved at 25 mg mL^{-1} in water:acetonitrile (1/1(v/v)) prior to injection. Separation was performed using a Vydac C18 column (250 × 22 mm, 10-15 μm) at a flow rate of 15 mL min^{-1} with a gradient from 80 to 100% of eluant B in eluant A, over 30 min (eluant A: 0.1% (v/v) TFA in water; eluant B: 0.1% TFA in acetonitrile). Separation was monitored at 280 nm. The purified peptides were lyophilized and characterized by MALDI-ToF MS using a Waters MALDI Micro MX machine, operated at 12 kV in the positive ion mode with an extraction delay time of 500 ns. The matrix solutions were prepared by dissolving sinapic acid (Sigma, 10 mg mL^{-1}) in water/acetonitrile (4:6) with 0.1% (v/v) TFA; the peptide (1 mg mL^{-1}) was mixed with matrix in a 1:1 ratio directly on a MALDI plate. The observed $[MH]^+$ mass was the same as the calculated value (2244Da).

Amino Acid Analysis to determine stereochemistry of singly hydroxylated (*D*)-leucine.

Computational studies on the simplified enzymatic reaction model indicated production of the (3*S*)-stereoisomer in the singly hydroxylated product of the (*D*)-leucine CA peptide oxidation. To test this, we carried out amino acid analysis (using the acid hydrolysis method described in Mantri *et al.*[18]) on the FIH catalysed product, comparing the results with those for racemic mixtures of (2*S*,3*R*)/(2*R*,3*S*) and (2*S*,3*S*)/(2*R*,3*R*) 3-hydroxyleucine. The mass chromatograms (m/z 318.1) imply production of the (2*R*,3*S*) product (assuming retention of (2*R*)- stereochemistry at C-2 (Supplementary Figure 5). Note these results also support the assignment of the singly hydroxylated product as C-3 hydroxyleucine.

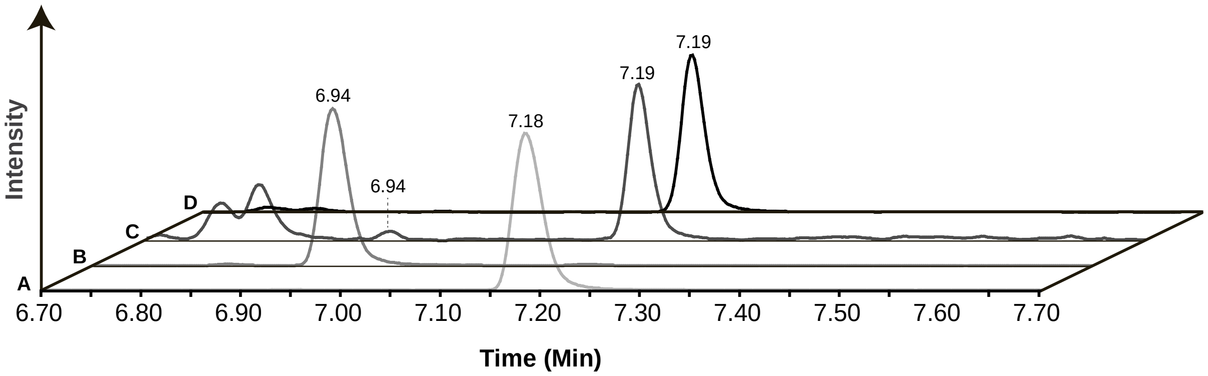

Supplementary Figure 5 Amino acid analysis of the CA_(*D*)-leucine substrate peptide after hydrolysis following FIH incubation imply that (*2R,3S*) 3-hydroxyleucine is formed. Traces correspond to results with **A**: synthetic (2*S*,3*R*)/(2*R*,3*S*) 3-hydroxyleucine (retention time 7.18 min); **B**: synthetic (2*S*,3*S*)/(2*R*,3*R*) 3-hydroxyleucine (retention time 6.94 min); **C**: 3-hydroxyleucine from the (*D*)-leucine CA peptide incubated with FIH (retention time 7.19 min), assigned as (*2R,3S*), and **D** hydrolysis of a synthetic (*2R,3S*) 3-hydroxyleucine CA peptide (retention time 7.19 min).

Supplementary References

1 Yang, M. *et al.* Substrate selectivity analyses of factor inhibiting hypoxia-inducible factor. *Angew Chem Int Ed Engl* **52**, 1700-1704, doi:10.1002/anie.201208046 (2013).

2 Chowdhury, R., Hardy, A. & Schofield, C. J. The human oxygen sensing machinery and its manipulation. *Chem Soc Rev* **37**, 1308-1319, doi:10.1039/b701676j (2008).

3 Becke, A. D. Density-Functional Thermochemistry 3. The Role of Exact Exchange. *J Chem Phys* **98**, 5648-5652, doi:Doi 10.1063/1.464913 (1993).

4 Lee, C. T., Yang, W. T. & Parr, R. G. Development of the Colle-Salvetti Correlation-Energy Formula into a Functional of the Electron-Density. *Phys Rev B* **37**, 785-789, doi:DOI 10.1103/PhysRevB.37.785 (1988).

5 Winn, M. D. *et al.* Overview of the CCP4 suite and current developments. *Acta Crystallogr D Biol Crystallogr* **67**, 235-242, doi:10.1107/S0907444910045749 (2011).

6 Winter, G. xia2: an expert system for macromolecular crystallography data reduction. *J Appl Crystallogr* **43**, 186-190, doi:10.1107/S0021889809045701 (2010).

7 McCoy, A. J. *et al.* Phaser crystallographic software. *J Appl Crystallogr* **40**, 658-674, doi:10.1107/S0021889807021206 (2007).

8 Adams, P. D. *et al.* PHENIX: a comprehensive Python-based system for macromolecular structure solution. *Acta Crystallogr D Biol Crystallogr* **66**, 213-221, doi:10.1107/S0907444909052925 (2010).

9 Elkins, J. M. *et al.* Structure of factor-inhibiting hypoxia-inducible factor (HIF) reveals mechanism of oxidative modification of HIF-1 alpha. *J Biol Chem* **278**, 1802-1806, doi:10.1074/jbc.C200644200 (2003).

10 Emsley, P. & Cowtan, K. Coot: model-building tools for molecular graphics. *Acta Crystallogr D Biol Crystallogr* **60**, 2126-2132, doi:10.1107/S0907444904019158 (2004).

11 Gomes, C. P., Metz, A., Bats, J. W., Gohlke, H. & Gobel, M. W. Modular Solid-Phase Synthesis of Teroxazoles as a Class of a-Helix Mimetics. *Eur J Org Chem*, 3270-3277, doi:10.1002/ejoc.201200339 (2012).

12 O'Donnell, M. J. & Polt, R. L. A Mild and Efficient Route to Schiff-Base Derivatives of Amino-Acids. *J Org Chem* **47**, 2663-2666, doi:Doi 10.1021/Jo00134a030 (1982).

13 Fraunhoffer, K. J. & White, M. C. syn-1,2-amino alcohols via diastereoselective allylic C-H amination. *J Am Chem Soc* **129**, 7274, doi:10.1021/ja071905g (2007).

14 Boyaud, F. *et al.* First Total Synthesis and Stereochemical Revision of Laxaphycin B and Its Extension to Lyngbyacyclamide A. *Org Lett* **15**, 3898-3901, doi:10.1021/ol401645m (2013).

15 Blaser, D. & Seebach, D. Benzyl (*R*) and (*S*)-2-tert-Butyl-5-Oxo-Oxazolidine-3-Carboxylate for Convenient Preparation of D-Threonine and L-Threonine Analogs from Aldehydes. *Liebigs Ann Chem*, 1067-1078 (1991).

16 Bonnard, I. *et al.* Total structure and inhibition of tumor cell proliferation of laxaphycins. *J Med Chem* **50**, 1266-1279, doi:10.1021/jm061307x (2007).

17 Soloshonok, V. A. *et al.* Biocatalytic Approach to Enantiomerically Pure Beta-Amino Acids. *Tetrahedron-Asymmetry* **6**, 1601-1610, doi:Doi 10.1016/0957-4166(95)00204-3 (1995).

18 Mantri, M. *et al.* The 2-oxoglutarate-dependent oxygenase JMJD6 catalyses oxidation of lysine residues to give 5S-hydroxylysine residues. *ChemBioChem* **12**, 531-534, doi:10.1002/cbic.201000641 (2011).
